# OCD Compulsions as Aberrant Integration of Uncertainty from Local Goals to Global Goals

**DOI:** 10.5334/cpsy.145

**Published:** 2026-08-10

**Authors:** Andra Geana, Christina L. Boisseau, Steven Rasmussen, Michael J. Frank

**Affiliations:** 1 Providence College, Providence, RI, US; 2 Brown University Carney Institute for Brain Science, Providence, RI, US; 3 Northwestern Feinberg School of Medicine, Northwestern, Chicago, IL, US; 4 Warren Alpert Medical School of Brown University, Providence, RI, US; 5 Butler Hospital, Providence, RI, US; 6 Brown University Alpert Medical School, Providence, RI, US; 7 Butler Hospital, Providence RI, US

**Keywords:** obsessive-compulsive disorder, compulsivity, computational modelling, OCD, learning, reinforcement learning

## Abstract

Recent years have seen unprecedented increases in compulsive behaviors, even in previously undiagnosed populations. Yet, despite their rising prevalence and the fact that compulsions—notably associated with obsessive-compulsive disorder (OCD), but also a key feature of other disorders such a hoarding or body dysmorphic disorder—significantly hamper daily function, the specific mechanisms of their formation remain unknown. Previous research holds mixed findings on compulsivity-linked cognitive deficits, and, while many accounts assume that compulsions aim to reduce anxiety, most individuals with anxiety do not develop compulsions—suggesting that the anxiety-reduction account is incomplete. We propose here a broader, uncertainty-resolving account of compulsivity, that bypasses the commonly-used, single-goal structure of many cognitive tasks to leverage the real-world hierarchical structure of goals—with “*local”, short-term subgoals* (e.g., “how do I clean my hands?”) in the service of *long-term, “global” goals* (“how do I stay healthy?”). In that framework, we suggest that compulsivity may reflect a deficit in integrating uncertainty from local to global goals, which still spares the basic circuitry of reward and learning. We tested 20 OCD patients and 20 healthy volunteers in a predictive inference task with a hierarchical structure of local uncertainty reduction in the service of global uncertainty reduction. Both groups learned the reward structure of the local environment based on observed data; however, only the healthy volunteers showed evidence of integrating learned knowledge at the local level to reduce uncertainty about the higher level of the task hierarchy. This indicates a potential mechanism for compulsions that relies on the inability to adaptively integrate, prioritize, and “toggle” among different local goals in the service of global goals.

## Introduction

The prevalence of obsessive-compulsive disorder (OCD) has increased worldwide in the last few years since the COVID-19 pandemic (Guzick et al., 2021), with contamination-related compulsions found at unprecedented levels even in previously undiagnosed populations(Tanir et al., 2020). Such compulsions, frequently manifesting as ‘checking’ or other elaborate routines to resolve uncertainty about the state of the world, force an individual to spend disproportionate time and effort engaged in a repetitive cycle, unable to break out and focus on other goals. For example, while a healthy person might choose to wash their hands after returning home from a ride on public transit, then (once their hands are clean) move on with usual daily life, a person with OCD might fear contamination even after handwashing. This fear might lead them to wash their hands again after touching their clothes or touching a doorknob; then, perhaps, they might choose to clean the doorknob, or mop the floor to ensure they did not track in contaminants – and then wash their hands again and again after each step.

Yet, despite rising prevalence and severe impact on daily life—OCD ranks among the leading causes of disability in the United States, and in up to 30% of cases, treatment (including combinations of medication and therapy) fails to significantly relieve compulsions (Silverman et al., 2022; Boisseau et al., 2017)—a mechanistic understanding of the root of compulsions is largely lacking. This is due in part to the variability in symptoms and high comorbidity with other disorders, and to the fact that OCD is unlikely to result from alteration of any singular molecular mechanism. For instance, some accounts frame compulsions from a learning-failure perspective, assuming deficits in the ability to correctly represent action-outcome links (Nielen et al., 2009; Fleming & Daw, 2017). Others suggest a lack of goal-directed control, proposing that OCD impairs the ability to act appropriately in pursuit of goals and reverting to habits (Nielen et al., 2009; Seow et al., 2021). Empirical data shows mixed evidence, with OCD individuals able to learn comparably to healthy volunteers on a variety of tasks, and goal-directedness failures inconsistent across tasks and domains (Zainal et al., 2023; Gillan et al., 2014; Soref et al., 2008; Gillan et al., 2011). Crucially, while anxiety is considered a vital factor in OCD, it is worth nothing that most people suffering from anxiety or intrusive anxious thoughts do not develop compulsions (Goodwin, 2022); thus, it is possible that mechanisms independent of anxiety contribute to the formation of compulsions, and elucidating these separate mechanisms is critical to our understanding of the disorder.

*Compulsivity as a Deficit in Uncertainty-Related Processes* While classical neurocognitive assessments of OCD often involve diagnostic interviews, questionnaires (including clinical scales) and self-reported measures (Rasmussen & Tsuang, 1986), observation from clinical settings suggests that many compulsive behaviors are aimed at reducing uncertainty, with the inability to do so ultimately perpetuating the irrational thoughts and behaviors (Hauser et al., 2017). Uncertainty plays a role in most learning and decision scenarios, from ordering dinner to choosing a job, and evidence across species shows that the ability to represent and reduce uncertainty is essential for optimal decision-making Wilson et al., 2014; Wilson & Niv, 2012; Frömer et al., 2021; Sewell et al., 2019). Failures to regulate uncertainty have been linked to maladaptive learning in a variety of neuropsychiatric disorders (Jacoby et al., 2023; Morein-Zamir et al., 2020; Vaghi et al., 2020; Gillan & Seow, 2020; Wise & Dolan, 2020). Individuals with OCD in particular exhibit a profound intolerance of uncertainty, and perhaps as a result, oversample in uncertain environments (Bucarelli& Purdon, 2016; Solway et al., 2021; Dar, 2004). However, uncertainty-driven computations remain relatively under-examined in the context of compulsivity, and it remains unclear whether patients simply show a heightened aversion to uncertainty *per se*. For example, patients might assign a more negative value to the prospect of the undesired outcome for a given level of uncertainty. Or, they might exhibit overall greater uncertainty due to disruptions in the computations needed to reduce it. Here, we consider the latter possibility.

It is possible that one source of mixed results in lab studies on learning deficits in OCD is the commonly-used task structure that assesses uncertainty about a single decision (e.g., learning the underlying reward structures of two independent options). In contrast, most real-world learning scenarios involve a hierarchy of goals, with “local” subgoals (e.g., “will washing my hands make them cleaner?”) in the service of broader, “global” goals (“how can I stay healthy?”). Under such framework, OCD patients may be able to resolve uncertainty about the outcomes of a local decision, but then fail to update the uncertainty about progress toward the global goal, due to disruptions in the mechanism responsible for integrating across timescales and hierarchical levels of abstraction. In line with this hypothesis, recent work (Solway et al., 2021; Dar et al., 2022; Fradkin et al., 2020) shows impaired transfer of information (in the form of explicit and implicit task-relevant memories) across repeated decisions. However, to our knowledge, the integration of information in the service of short-term and long-term goals has never been tested in OCD; a mismatch in the integration of these parallel learning processes into global goals can produce compulsive behaviors.

Using a predictive inference task with a hierarchical learning structure, we tested this hypothesis in a sample of individuals with OCD and age-matched controls. While OCD patients showed intact local learning of stochastic outcomes, they exhibited impaired ability to leverage this learning to update their beliefs about global structure. We propose that a mismatch in the integration of these local learning processes into global goals can produce compulsive behaviors, while preserving the ability to locally reduce uncertainty.

## Methods

### Participants

Participants were recruited through the OCD Research Program at Butler Hospital. Out of the 463 participants that were phone screened for initial eligibility, 55 (8.4%) met criteria for in-person evaluation for final determination of eligibility. To be included in the OCD group participants were required to be adults age 18–55, meet DSM-5 criteria for OCD, and identify OCD as their primary psychiatric concern. Exclusion criteria for the OCD group included past month substance use disorder or report of lifetime bipolar or psychotic disorders. Healthy controls were free of current psychiatric disorders.

Of the 55 individuals that past the initial phone screen, 4 were ruled out of the OCD group because of subclinical symptoms, 3 were ruled out of the healthy control group due to the presence of psychopathology or unreliable report and an additional 5 did not show up to the in-person assessment. Thus, the final sample contained 20 participants with OCD and 23 healthy controls. The groups were matched on age and general intellectual ability as measured by the National Adult Reading Test-Revised (NART-R; Blair & Spreen, 1989). All participants gave informed, written consent approved by the Institutional Review Board. They were compensated $80 dollars for approximately four hours of their time.

### Measures

Psychiatric diagnoses were made by a trained postdoctoral fellow based on DSM-5 criteria using the Structured Clinical Interview for DSM-5 (SCID-5; First et al., 2015). Severity of obsessive and compulsive symptoms was assessed using the rater-administered Yale-Brown Obsessive-Compulsive Scale (YBOCS) and Symptom Checklist (Goodman et al., 1989). Depression and anxiety symptoms were measured using the self-report Beck Depression Inventory (Beck et al., 1996) and Beck Anxiety Inventory (Beck et al., 1998). Finally, intolerance of uncertainty was measured using the self-report Intolerance of Uncertainty Scale (IUS, Buhr & Dugas, 2006).

#### The Archer Task

**Figure 1:**
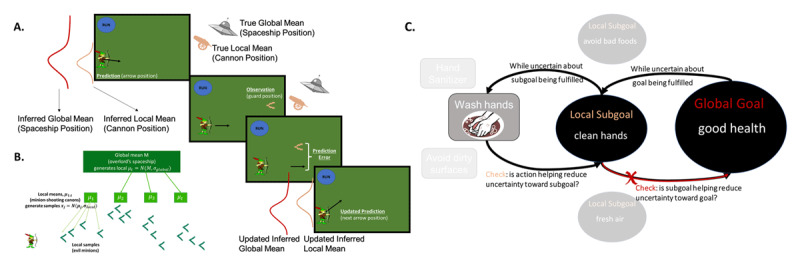
The Archer Task. **A.** Example trial, starting with **prediction** (player’s aim with the arrow), followed by the **observation** of a data point (minion) from the local distribution (canon), and the **updated prediction** (adjusting aim for next trial). The hierarchical structure of the task, with the two distributions governing minions and canon positions are show on the side of the screen. Panel **B.** shows a graphical representation of the hierarchical Gaussian distribution, with the global distribution centered on a global mean (M) governing the distribution of local means, which in turn determine the position of each minion the player observers. **C.** Conceptual representation of how uncertainty can be used in the service of integration of local into global goals, and how the inability to integrate (bottom right-side arrow, red), even accompanied by intact ability to reduce uncertainty (left-side circuit) can lead to becoming “stuck” in local repetitive-action cycles.

We developed a new predictive inference task, building upon the basic structure of previous tasks designed to challenge dynamic learning in uncertain (stochastic and volatile) environments (Nassar et al., 2012; Geana et al., 2016). We modified this task (Figure 1) to include a hierarchical structure, with local outcomes generated through an overarching global structure. Players had to aim arrows to shoot enemies, the locations of which were hidden, but could be inferred from sequential observations.

The task was framed as a game in which players must defeat an “evil overlord” by destroying his spaceship (located at an unknown position on the screen). The spaceship sends “cannons” to attack the archer, with each cannon launching up to thirty “minions” (*local samples*), sequentially. To introduce uncertainty, each minion’s position is drawn from a Gaussian distribution, whose *local mean* is centered on the position of the cannon. Each cannon’s position is, in turn, launched by the spaceship with stochastic variations around the *global mean*. This hierarchical structure ensured that observing each minion’s position can reduce uncertainty simultaneously on two levels. At the *local* level, the position of an individual minion provides a sample of evidence about the position of the current cannon, allowing the player to adjust their subsequent aim. With more samples they should have a more certain estimate about the cannon position (the local mean) and thus progressively adjust less with each stochastic outcome. At the *global* level, the player can use their updated estimate of the current cannon’s position to infer spaceship position.

The players earned a small reward (1 point) for accurately aiming arrows to hit each minion’s position, and a large reward at the end of the game (60 points) for accurately firing an arrow at the spaceship (they were instructed about this payoff structure during pre-task instructions as well as during the training games). At the end of the task, earned points were converted to a “bonus” reward of up to $5.

#### Trading off local and global reward

A vital aspect to the task was that players had a limited number of 210 arrows. They were made aware of this limit at the beginning of the task, and during training; however, they did not see the remaining number arrows once training was completed and the task began. When all but one arrows were used up, the player would immediately encounter the spaceship and have one chance to fire at it. At that point, a more accurate estimate of the spaceship’s location would translate into a better chance of earning the significantly higher global reward. On the other hand, each local canon could fire up to 30 minions. By sampling a series of minions, the player can be relatively confident about the canon’s location and then accumulate local rewards (r = 1 point each) relatively easily (i.e., by aiming at the mean of their previous observations). To reflect this trade-off, on each trial, players were given the chance to decide whether to “stay” and face more minions from that cannon (thus improving their local estimate) or “leave” and see a new cannon (i.e., sample more from the global structure and improve their global estimate).

Players obtained a reward based on the accuracy of each trial’s aim. Accuracy was measured by the difference in y-axis position between the player’s aim and the minion’s position; the y-axis was split into 200 intervals measured relative to the size of the experimental screen (so in relative screen size units rather than absolute centimeters or pixels; all participants completed the task on the same screen, so they all experienced the same accuracy calculations. The task was designed so a ‘hit’ on screen occurred if the aim deviation from the minion position fell below the standard deviation of the local generative distribution *σ_Local_*. This coincided with a small window of about 25px around each minion’s y-axis position. Given the task structure, the chance for earning rewards in each game was indeed maximized by aiming at the position of the local cannon – but participants only earned points if they actually “hit” the minion.

In sum, to maximize total reward for the task, players had to learn both each local mean (i.e. learn where each cannon is, to accurately predict where the minions will come from) and the global mean (i.e. spaceship location) that generated all these local means. The speed of their learning on both these processes changed the optimal point for continuing or quitting each local game (see modelling below for formalizing this tradeoff).

All participants played the same number of trials (210), but due to the player-made decision to stay or leave, the number of games (waves of local minions) ranged from a minimum of 7 to a maximum of 15, with an average of just under 10 games. The majority of learning analyses as well as model fits presented below included only complete games, excluding all games during which participants ran out of arrows mid-game. (This translated to an average of around 190 trials per participant in the healthy volunteers group, who were more likely to have left earlier games and thus ran out of arrows in the middle of the last game more frequently. Participants in the OCD group did not often use the option to leave and thus nearly always had seven full games of maximum length).

#### Computational Modelling

For a deeper quantitative and mechanistic understanding, we take a computational psychiatry approach (Huys et al., 2016), mixing theory-driven and data-driven methods to predict behavior based on individual inferences about the structure and likelihood of different outcomes in the world. Assuming a hierarchy of long-term (global) and short-term (local) learning goals, our computational model of the archer task allows us to test to what degree participants performed parallel updating in the estimation of local and global uncertainty, and how they integrated the two decision problems to solve the task. All modeling and statistical analyses were conducted in MATLAB, using the Statistics and Machine Learning toolbox.

### Uncertainty-Updating Model

On each trial (*t*) of each game (*g*), the model uses observed minion positions (*x*) to estimate the position of that game’s cannon (*μ*_g,t_), using Bayesian updating of the local means based on observed samples:


Equation 1 $$\left(\mu_{\text{g},\text{t}}|x_{\text{g},1:\text{t}}\right)\;\;\;=\;\;\;\text{N}\left(\mu_{\text{g},\text{t}},\sigma_{\text{g},\text{t}}^{2}[M_{\text{g}-1}/\sigma_{\text{G},\text{g}-1}^{2}+\;\left(t\cdot {\bar{x}}_{\text{g},\text{t}}\right)\;\;/\;\sigma_{\text{Local}}^{2}],\sigma_{\text{g},\text{t}}^{2}\right)$$

where the posterior local variance *σ*^2^_g,t_ is recursively defined as $\sigma_{\text{g},\text{t}}^{2}={\left[1/\sigma_{\text{L},0}^{2}+\text{t}/\sigma_{\text{Local}}^{2}\right]}^{-1}\cdot {\bar{x}}_{\text{g},\text{t}}$ is the sample mean of the observed minion positions *x*_g,1_, *x*_g,2_, …, *x*_g,t_ up to current trial *t* within game *g*. *σ*^2^_g,t_ represents the posterior local variance, *σ*^2^_L,0_ is the prior local variance, and *σ*^2^_Local_ represents the local observation variance (derived from the pooled variance around the inferred means *μ*_g,T_ of all previous *g*–1 games). For the first game, *μ*_0_ is initialized randomly to a position close to the middle of the screen (the same as the initial prior for the spaceship’s position, or the global mean), and *σ*^2^_L,0_ is initialized randomly between 5 and 10. For the other games, the model uses estimates from the global mean distribution. The maximum game length, *T*, is set to 30 for this experiment; the local reward *r* is set to 1 point, and the global reward *R* is set to 30 points.

**Figure 2:**
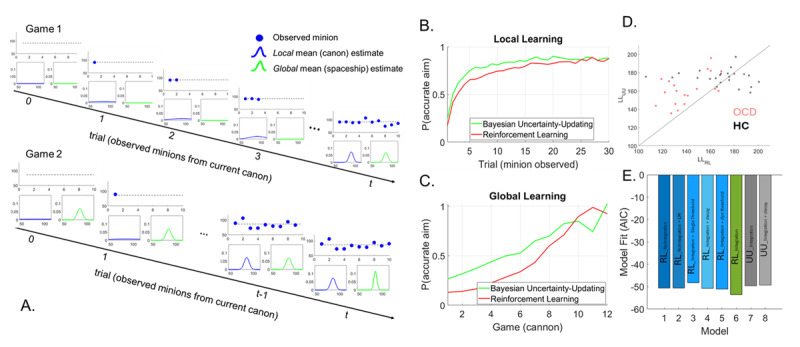
Models Integrating Local and Global Uncertainty. **A. Uncertainty-Updating Bayesian model:** Example of local observed samples (blue filled circles) changing the estimate of the local mean (blue curves) on each trial of the game; at the end of a game, the final estimated local mean is used as one sample to update the estimate of the global mean (green curve). The local mean estimate resets between games. The global mean estimate carries from game to game, becoming updated at the end of each game. **B.** Simulation showing how under the model assumptions, more observed local samples translate to a better estimate of the local mean and improved aim accuracy (i.e., local learning throughout a game) for both Bayesian Uncertainty-Updating (green) and RL (red) models. **C.** Simulated model behavior showing improved aim on the first trial of each new game, i.e. global learning, when the local mean estimates are integrated into estimating the global mean for both models. **D.** Per-subject likelihoods for participants in either group better fit by either the UU or RL model. **E.** Model comparison using the AIC.

The model tracked its own uncertainty about the estimated local means as the variance of the estimated local mean distribution, with no added noise. This assumption was considered reasonable in the context of the task given the relatively low generative variances and simple visual task demands: while adding noise to the estimated mean accurately reflected task performance, additional uncertainty in the estimates of sample variance was not considered to bias the decision significantly; thus the noise generally associated with imperfect Bayesian perceptual inference in humans (e.g. Hermans et al., 2008) was integrated into our present model at the aim decision step, as noise parameter *ɛ* (see below). With more observed samples (larger *t* in a game), that estimated local uncertainty *σ*^2^_g,t_ decreased proportionally to 1/*t* (see Figure 2A), and the probability of accurate aim on each subsequent trial increased (see Figure 2B). (While this is not always necessarily reflective of uncertainty in estimating generative variance, it was consistent with the task structure here, given the generative parameter settings).

At the end of each game, the model updated its estimate of the spaceship’s position in a similar manner, using the last estimate of the game’s local mean, and also tracked its uncertainty regarding this global mean estimate:


Equation 2 $$p\left(M_{\text{g}}|\mu_{\text{g},\text{t}}\right)=\;\;N\left(M_{\text{g}},\sigma_{\text{G},\text{g}}^{2}[M_{\text{g}-1}/\sigma_{\text{G},\text{g}-1}^{2}+\mu_{\text{g},\text{t}}/\sigma_{\text{Local}}^{2}],\sigma_{\text{G},\text{g}}^{2}\right)$$

where the posterior global variance *σ*^2^_G,g_ is defined as:


Equation 3 $$\sigma_{\text{G},\text{g}}^{2}=\;\;\;{\left[1/\sigma_{\text{G},\text{g}-1}^{2}+\;\;1/\sigma_{\text{Local}}^{2}\right]}^{\;-1}$$

Where *σ*^2^_G,g–1_ and *M*_g–1_ represent the variance (uncertainty) and mean estimate of the global distribution from the previous game *g*–1, ensuring recursive accumulation of global knowledge across successive games. Note that for the model shown in Equations 1 and 2, rather than fully integrate, we used an empirical Bayes approach to simplify the estimation: specifically, we assumed that for each new local process, the prior mean *μ*_0_ was drawn from the global process and the prior *σ*^2^_L,0_ was inferred from the observations of all previous processes. Similarly, this pooled variance was used as an estimate for the hyperparameter *σ*^2^_g_ (rather than maintain a probability distribution over the variance and marginalizing it out or using an MCMC sampler). The rationale for this simplification rested partially on the constraints on plausible mental computation and partially on keeping the model more easily adaptable to changes in task design that would require significant shifts in a fully-Bayesian inference (e.g. adding multiple blocks with similar hierarchical structure). The derivation of the equations followed these assumptions.

After the first game, the current estimate of the spaceship’s position (the global mean) served as prior for the location of the cannon in the next game. The optimal place to aim upon encountering each new cannon (before observing any minions it sends) is this estimate of the spaceship position. Indeed, accurately learning the global distribution across games should lead to a gradual improvement in performance on the first trial of each new game (Figure 2C) – a key signature of integration of evidence from local to global levels. Conversely, failure to accurately integrate local information from each game to update the estimate of the global mean would lead to no performance improvement on the first trial of each new game (and thus players might aim randomly, or based on the last estimate of the local mean only).

The model assumed that players would aim at the current estimated local mean on each trial (based on the inference processes above), with a noise parameter ɛ. Under that assumption, it then predicted the decision whether to *stay* or *leave*, based on current uncertainty about local and global means. The values for the two actions were computed as follows: *stay* was associated with reduction in local uncertainty (which decreased as the number of trials in a game increased, leading to diminishing marginal returns on information with longer game times) and increase in the probability of obtaining local rewards. Staying led to no change in global uncertainty (as the model updated the global estimate only at the end of a game). Conversely, *leave* was associated with an increase in local uncertainty (which reset at the beginning of each game to the current estimate of $\sigma \frac{2}{G}$) and a decrease in estimated local reward, but also with a reduction in global uncertainty, and thus a small increase in the probability of obtaining the larger reward at the end of the task. Thus, the local and global reward estimates traded off on each trial in each game, depending on the uncertainties in the current estimates of the local and global means.


Equation 4 $$V_{\text{stay}}\propto \left(T-t\right)\cdot P_{\text{hit}}\left(\sigma_{\text{g},\text{t}}^{2}\right)+R\cdot P_{\text{hit}}\left(\sigma_{\text{G},\text{g}-1}^{2}\right)$$

Equation 5 $$V_{\text{leave}}\propto \left(T-t\right)\cdot P_{\text{hit}}\left(\sigma_{\text{G},\text{g}}^{2}\right)+R\cdot P_{\text{hit}}\left(\sigma_{\text{G},\text{g}}^{2}\right)$$

Where *t* refers to the current trial in the current game (out of a maximum total *T* = 30 trials in the maximum game length) and *R* is the total reward for correctly hitting the overlord’s spaceship at the end of the task (*R* = 30 points in the current game settings). *P*_hit_(*σ*^2^) denotes the probability of a successful hit, which, given the assumption that participants aim at the current inferred local mean on each trial, depends on the uncertainty around that estimate (i.e. the current estimate of the local variance). In other words, the odds of hitting a minion are maximized by aiming at the current inferred local mean, but larger variance equates to lower probability of a hit, as each observed minion will be close to, but not exactly at, the mean. While the size of each minion allowed some flexibility in aim around the mean; *P*_hit_(*σ*^2^) captures the mathematical dependency between current estimated local variance and the probability of actually hitting the minion even after learning the mean.

Lastly, the model assumed that participants decided what to do using a softmax rule comparing these two values (stay vs. leave), with inverse temperature parameter *β*. Variants of the model that included random choice noise were also considered (for both the Uncertainty-Updating and the RL model below), but did not perform as well in model comparison (see Figure 2E).

### Reinforcement Learning Model

The uncertainty-updating model assumes Bayesian computations of the estimated mean and uncertainty about the mean on each trial (for local means) and at the end of each game (for global mean), as well as forecasting probabilities of reward under different conditions (Stay or Leave) on each trial. While ample evidence exists of the brain engaging in Bayesian updating (Wise et al., 2019; Yon & Frith, 2021; Loeb & Fishel, 2014), including in the learning and decision-making domain (Poudel et al., 2017; Fleming & Daw, 2017), such a model is usually expensive in computational resources (and, depending on how it is implemented, may or may not also require storing full distributions rather than solely parameters – although our current model above is simplified to some extent and lower on memory load) and requires perhaps more resources than participants are able or willing to expend on the task. Reducing or simplifying the Bayesian components (as, e.g., shown in the UU model above) is one way to preserve the inferential advantages of a Bayesian framework while mitigating the potential computational expenses; however, alternative strategies may also avoid inference computations entirely and instead learn the structure of the environment and the reward-maximizing policies via trial-and-error.

For instance, a less demanding strategy might entail learning the correct place to aim via trial-and-error, and tracking the change in prediction error (PE, the difference between where the player aimed and where the observed minion appeared on screen) across trials. Previous research strongly indicates that trial-to-trial changes (specifically, the amount of reduction) in prediction error are indicative of the quality of learned information and the need to learn more (Frömer et al., 2021 Sewell et al., 2019). Thus, a model tracking these changes could heuristically estimate, based on thresholds for how much the PE is being reduced, the value of local and global learning, and use that strategy to decide whether to Stay or Leave.

Our reinforcement learning (RL) model updates the local means on each trial based on prediction error PE, with a local learning rate *α_L_*, as


Equation 6 $$\mu_{g,t}=\mu_{g,t-1}+PE*\alpha_{L}$$

Where PE is the prediction error observed on each trial, expressed as


Equation 7 $$PE_{t}=\left(x_{g,t-1}-\mu_{g,t-1}\right)$$

where *x_g,t_*_–1_ is the most recent observed minion position. The global mean M is similarly updated, with a global learning rate *α_G_*.


Equation 8 $$M_{g}=\mu_{g-1}+PE*\alpha_{G}$$

Equation 9 $$PE_{g}=\left(\mu_{g,t}-M_{g}\right)$$

For this model, the global means were updated at the end of each game g. Multiple potential ways to include the learned global structure into local predictions were tested; based on model fits and in order to match the assumptions of the Bayes Uncertainty-Updating model, the current RL model similarly integrates the learned global structure into the aim decision of the first trial of each new game.

The decision whether to stay or leave a current game is made heuristically, based on the change in tracked local and global prediction errors Δ*PE_L_*, Δ*PE_G_*, and local and global PE-thresholds *γ_L_, γ_G_*, as:


Equation 10 PStay={0,if ΔPEL<γL and ΔPEG>γG1,otherwise00000000000000000000                with ΔPEL=PEt−PEt−1

As the model learns the underlying distribution of each local game, the local prediction error decreases, and, crucially, the change in prediction error Δ*PE_L_* also decreases; while the local and global PE settle near the level of the generative-process standard deviation (and so never decrease to 0), average Δ*PE_L_* can reach values close to 0 once the model has learned where to aim. A low value of Δ*PE_L_* is indicative that all relevant information from the local game has been learned, and it would be useful to leave and begin a new game. Conversely, a high value for Δ*PE_L_* indicates that there is still relevant information to be learned about the global structure. Thus, low local PE change and high global PE change indicate that, from an optimal learning perspective, leaving is the correct decision.

A class of RL models that did not include the integration component of local and global uncertainty were also tested; these no-integration models similarly updated local means based on observed minions, but either ignored the known task structure and did not include global mean computations into their policy, or did not update the global mean based on local means (instead updating it as a weighted mean of the most recent observations). The no-integration RL models also did not take into account the decrease in prediction error for stay-or-leave decisions, thus ignoring the local-reward-vs-global-information tradeoff.

### Model Comparison

Both the Bayesian Uncertainty-Updating and the RL model were simulated and fit to all participants’ data. Figure 2A shows the Bayesian model update process. Figure 2B and 2C show local and global learning for both models; in both, the predictions for local and global accuracy improving with increased numbers of observations are similar, but they occur through different underlying architectures (Bayesian inference vs. tracking trial-to-trial reductions in Prediction Error).

Both models were fit to participants’ data using MATLAB’s fmincon function, with the fits iterated over N = 5 different starting points. The RL model fitting better in the majority of cases for both groups. Figures 2D and 2E show model fits.

## Results

As shown in Table 1, there were no significant differences between the groups on age, general intellectual ability, gender, or minority status. The OCD group reported a moderate level of OCD symptoms (*M* = 21.7, *SD* = 5.1), mild depression (*M* = 15.3, *SD* = 1.6), moderate anxiety (*M* = 16.7, *SD* = 11.7) as well high levels of intolerance of uncertainty (*M* = 79.6, *SD* = 23.3). Fifty-two percent of the OCD participants reporting taking psychiatric medication, including serotonin reuptake inhibitors (n = 12), and benzodiazepines (n = 1).

**Table 1:** Sample demographics.


	OCD	HC	*P*

Age	29.4 (9.4)	30.5(10.1)	.702

Female, n (%)	16 (80)	17 (81.0)	.384

Estimated Verbal IQ	120.5 (7.8)	119.2 (6.9)	.567

Employed, n (%)	14 (60.9)	14 (66.7)	.690

Ethnic or racial minority, n (%)	7 (30.4)	7 (33.3)	.837


### Local Learning

**Figure 3:**
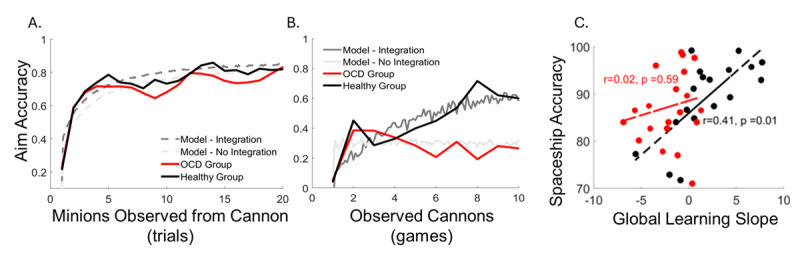
Local Learning and integration of local into global learning. A. Both the control group (black line) and the OCD group (red line) reduced their uncertainty about canon position with more minions observed from one canon. The grey dotted lines show model predictions for this local learning with (RL model described above) and without (the no-integration RL model described above) integration of local into global estimates. Note that all models tested showed similar learning patterns (with different slopes or asymptotes) on local games; the models shown here are the RL models with parameters that reflect the average group values returned after model fitting for each group. B. The control group (black line) shows improving aim on the first trial of new games, across the task. The OCD group (red line) does not. The grey lines show predictions from the same RL models that either integrate information from local to global (RL model, dark grey) or do not integrate that information (no-integration RL, light grey). C. Global accuracy correlates with the slope of global learning only in the HC group (black markers) and not in the OCD group (red markers).

Both the group of patients (OCD) and the group of healthy volunteers (HC) learned the position of the canon in each game, gradually improving their aim throughout the duration of the game. A mixed ANOVA looking at average aim accuracy across Time (20 trials) and Group (OCD and HC) showed a significant main effect of Time, with accuracies later in the game significantly higher than earlier in the game (*F*(19,779) = 54.04, *p* < 0.01), and a marginal effect of group, with the average accuracy overall in a game slightly higher for the healthy volunteers than the OCD patients (*M_HC_* = .69, *SD_HC_* = .15, *M_OCD_* = .655, *SD_HC_* = .12, *F*(1,41) = 4.43, *p* = 0.042). There was no significant interaction, suggesting that both groups learn similarly across the trials of a game (*F*(19,779) = 1. 043, *p* = .12; Figure 3A).

### Integration of Local into Global Learning

We examined participants’ ability to integrate the information they gained in the local games into their estimate of the global mean by looking at their aim accuracies on the first trial of each new game. As the position of their archer was reset to a random point at the beginning of each new game, and the local means of all games were independently sampled from the global mean, any gain in accuracy on the first trial across games was likely due to participants applying what they learned of the global structure to select a better prior for their estimate of the yet-unseen local structure.

As predicted by our conceptual model of OCD as a disruptor to the local/global integration of information, only the healthy volunteers group showed significant improvements in the first trial across games, while the OCD group had no effect. (Figure 3B; *M_HC_* = .48, *SD_HC_* = .07, *M_OCD_* = .29, *SD_HC_* = .072, significant Group × Time interaction, *F*(19,342) = 2.01, *p* = 0.007).

We also examined the accuracy of the participants’ guess on the last trial of the task (when they gave an explicit estimate of the global mean by aiming a last arrow at the spaceship). Consistent with reduced global learning, the HC group was slightly more accurate in their estimates (*M* = 7.73, *SD* = 3.50) than the OCD group (*M* = 10.07, *SD* = 3.95; two-tailed unpaired t-test, *t*(42) = 2.06, *p* = .045). Moreover, the HC group showed a significant correlation between the amount of integration of local into global learning (as measured by the slope of the global learning curves in Figure 3B) and their accuracy on that final global estimate (*r*(19) = 0.419, *p* = 0.01). This correlation was not present in the OCD group (Figure 3C; *r*(19) = 0.02, *p* = 0.59).

It is worth nothing that this correlation between global learning and spaceship accuracy makes sense under the assumption that estimated global means play a role in determining aim on the final (spaceship) trial. Absent that assumption, we would expect no correlation between the two. Strategies that do not integrate learned information about the global mean into the final aim – including random aim, aiming at the last observed minion, a noisy mean of all observed minions, and others – in addition to potential variability among participants in their chosen strategy, would decouple the global learning from the final trial accuracy.

### Local Learning, Global Learning, and Stay/Leave Behavior

**Figure 4:**
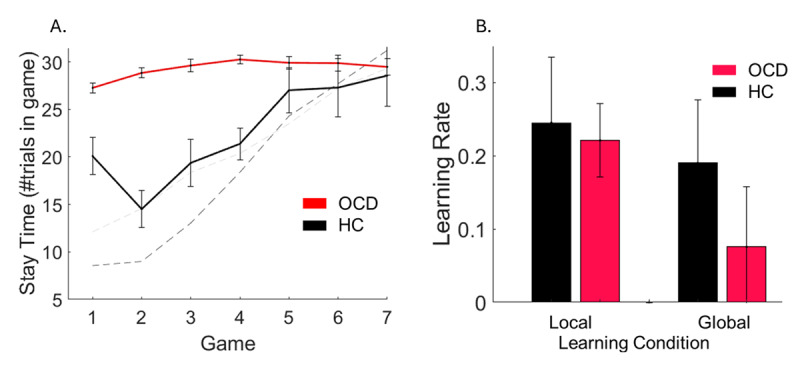
Behavior & Model parameters reflecting global learning. A. Stay times (how many guards attempted per game) increase with number of games in the HC group (black solid line), but not in the OCD group (red solid line). Grey dotted lines represent optimal stay times under different model frameworks (light-grey: RL model; dark grey: uncertainty-updating model). B. Fit model parameters for local and global learning rates in the RL model.

We examined trends in participants’ stay times (i.e., how many trials they spent in a game before pressing the RUN button to leave) over the course of the task as another behavioral indicator of their ability to integrate local and global learning. Both models predicted shorter stay times in earlier games, when there was higher uncertainty about the global structure, and progressively longer stay times in later games, as more local cannons were observed and thus the uncertainty about the spaceship position was reduced (see Figure 4A, dotted lines).

Participants in the HC group also followed this trend, with average stay times in early games (*M_game_*_1_ = 22.6, *SD* = 7.4) significantly different from later games (*M_game_*_7_ = 27.4, *SD* = 5.82; repeated – measures ANOVA, *F*(6,228 = 4.88, *p* < 0.01)). In the OCD group, however, stay times did not show the same pattern, with early and late games both showing significantly longer stay times compared to the HC group (*M_game_*_1_ = 28.2, *SD* = 4.00, *M_game_*_7_ = 29.9, *SD* = 0.44, *F*(1,228) = 2.85, *p* = 0.01. See Figure 4A, solid lines).

Model fits for learning rates and thresholds from the RL model showed significant differences in parameters for local and global learning, with global learning rates for the OCD group significantly lower than rates for the control group (*M_Global_HC_* = 0.19, *SD* = 0.08, *M_Global_OCD_* = 0.07 *g, SD* = 0.07, *t*(19) = 4.80, *p* < 0.01; see Figure 4B). Local learning rates, as well as thresholds for local and global learning were not significantly different (*t*(19) = 0.85, *p* = 0.4).

### Potential Alternative Strategies for OCD Group’s Global Learning

To better understand the potential strategies and differences in the local and global learning performance of the OCD group, we tested a series of potential alternatives for their learning, specifically focusing on their strategy for aiming on the first trial of a new game.

We first ran general linear regression model examining the impact of various measures on the first-trial aim position. These measures included: the position of the last observed minion in the previous game (LO), the aim on the last trial of the previous game (LA), the aim on the first trial of the previous game (LFA), the random starting position the task automatically set the archer to on the respective current game (SP), the mean of last three or the last five observed minion positions in the previous game (LM3/LM5), as well as a weighted mean of all observed minion positions so far (with the weight decayed as a function of how many trials back they were observed, AM). Figure 4A shows the results, with none of the regression coefficients significant predicting the first-trial aim.

We also examined the aim-noise parameter ɛ for differences between groups (Figure 4B). A two-way unpaired t-test showed a marginal difference (*t*(42) = 1.95, *p* = 0.057), with the average aim noise slightly larger in the OCD group (*M_HC_* = 6.92, *SD_HC_* = 1.19; *M_OCD_* = 7.56, *SD_HOCDC_* = .098). The temperature parameter in the softmax did not differ significantly between groups (*t*(42) = 0.95, *p* = 0.38).

To better understand potential differences in aim strategies, we also compared the individual variability in aim across games in the two groups. As participants observe more canons, if they integrate information from local games into their estimate of global structure they should gradually show reduced variability in their aims (as their accuracy improves and they should also be less sensitive to occasional outlier minion positions). Figure 4C shows this pattern of decreasing aim variability in the HC group, but not in the OCD group.

## Discussion

Our results suggest the OCD group shows preserved ability to reduce uncertainty at a local, but not global level. This is consistent with existing literature regarding oversampling/overfocusing in OCD (Hauser et al., 2017; Harkin & Kessler, 2012; Hermans et al., 2003); however, our task and model further explain conflicting findings on whether OCD patients can accurately represent and reduce uncertainty (Jacoby et al., 2023; Morein-Zamir et al., 2020). Specifically, we propose based on this data that, while the circuitry of reward and uncertainty computations may function to a large degree as it does in a healthy population—as shown by the intact learning at the local level in each game (Figure 3A), the mechanism responsible for integrating across timescales and generalizing from local to global goals is impaired in OCD.

Further work is needed to precisely characterize the degree and neural bases of this impairment. One possibility involves disrupted reinforcement learning (RL) within the nested hierarchical structure among corticostriatal circuits, where more anterior frontal regions represent global abstract rules that contextualize action selection in posterior circuits involved in achieving subgoals (Frank & Badre, 2012; Collins & Frank, 2016; Balleine et al., 2015). While RL might be intact at any individual level, impairment in cross-level integration would hamper abstract goals even if the subgoals have been achieved. Another possibility includes an aversion to uncertainty in identifying hidden task states at the level of orbitofrontal cortex (Schuck et al., 2016).

Crucially, our task helped identify differences in the ability to process information across local and global levels simultaneously: the OCD group did not show evidence of employing learned global structure to improve local first-trial aims across games (Figure 3B), nor did they adjust aim variability in later games as the model using information-integration would predict (Figure 4C). Model simulations show this behavior is consistent with a lack of integration across games of learned local structure in the service of learning the global structure; it is also consistent with the failure to employ the knowledge about the global structure to further guide or refine behavior at the local level.

It should be noted that while both groups learned the task at the local level, the OCD group may have shown some costs in the local learning performance of the lack of integration of global priors (Figure 3A). A potential mechanism for that small difference may be a benefit in the starting point of each new game associated with integrating new information about the global structure into the estimate of new local targets. However, the present task did not have sufficient data points to fully differentiate between that potential benefit of global learning on local trials and other mechanisms, such as increased noise, smaller/more precise trial-to-trial aim adjustments, memory decay or lower confidence in memory. While the potential differences in learning between the groups at the local level remain of interest, for the scope of this paper, we focused primarily on the binary distinction between improved learning on the local level and lack of evidence for global learning in the OCD group.

Interestingly, the performance of the OCD group on the last trial of the task, when participants explicitly estimated the global mean by aiming an arrow at the spaceship, was above chance and only slightly worse than that of the HC group. The lack of improvement on the first trial of each new game (Figure 3B), as well as the lack of correlation between the spaceship aim and the change in first-trial aim across games (Figure 3C) suggest that the OCD group did not rely on integration of local information learned throughout the task to estimate the spaceship position. Due to relatively small variances in the generative distributions, it is possible that they could still achieve accuracy on the spaceship trial by treating it as another “local” game. The regression in Figure 4A did not find first or last aims of the previous local game a reliable predictor for aims in the OCD group; however, as there were over 200 trials before this last trial, it is possible players were relying on other parts of their observed local structure that were not tested in the regression model.

**Figure 5:**
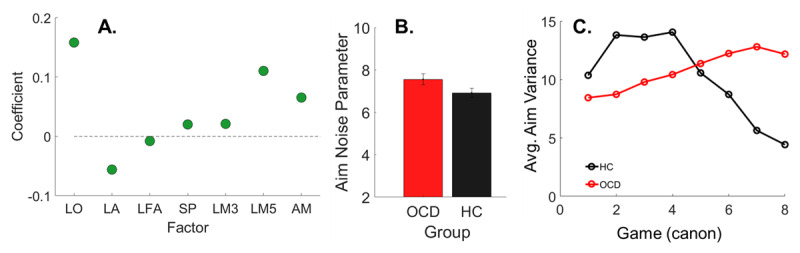
Alternative strategies to integration of local into global learning for the OCD Group. **A.** No significant coefficients from a general linear regression model when the last (or last few) observed minions (LO, LM3, LM5), the first/last aims on the previous game (LA, LFA), the current archer position (SP), or a weighted mean of all observed minions (AM) were used as potential predictors. **B.** The model parameter governing the noise in aim is slightly higher for the OCD (red bar) than the HC (black bar) group. **C.** The HC group (black) reduces aim variability across games; the OCD group (red) does not.

The practicality of maintaining a meaningful dependency between minion waves while sufficiently separating the waves visually on the computer screen, while at the same time keeping all stimuli sufficiently large as to remain accessible and engaging for the players, poses a potential limitation to the study design. As the screen could feasibly be split into about 200 aim intervals while preserving reasonable visibility in the game, the design question of calibrating variances, minion location constraints, minion sizes and other design parameters was carefully considered and tested over several small-scale pilot studies to calibrate these to the best possible extent to suit our goal of comparing two strategies: one that takes into account learned information about the global structure of rewards (and thus optimally aims at the current estimated global mean), and one that does not. For the latter, several aim strategies may be included, as discussed above: aiming at random, aiming at the last minion, and other options as shown in Figure 5A. While the relatively low number of games per participant and the design constraints made it more challenging to fully compute the cost of suboptimal strategies (partly due to the fact that it was still possible – though less likely – to hit the spaceship on the final trial without uncertainty integration), the strategy of aiming at the current inferred mean on the first trial of each new game remains mathematically optimal, and, we argue, intuitive in the context of the task’s narrative story.

While our task design and model allowed testing for local-to-global integration and how information was used simultaneously in the service of multiple goal levels, we could not test, for instance, whether the OCD group integrated local information throughout the task to estimate the global mean but did not apply their knowledge of the global structure to aid performance on local games. This latter would be consistent with findings of impaired transfer of information in OCD (Fradkin et al., 2020). It is also possible that, if this group did to some extent use observed minions to estimate the spaceship’s position, they did not perform that computation until the last trial, when instructions explicitly required it (all participants saw a screen warning that they were about to confront the spaceship). There is evidence that OCD patients perform implicit learning tasks better with additional explicit instruction, and that explicit task demands reduce observed deficits in OCD groups (Soref et al., 2018; Barzilay et al., 2022). Our task instructions explicitly mentioned the hierarchical structure (and the benefit of learning the global mean), and all participants reported in post-task debriefings that they were aware of the structure; but there were no reminders through the task of the implicit global learning necessary for optimal performance (as done, for instance, in Soref et al. (2018)). It is therefore possible that the OCD group was initially aware of the need for tracking the global and local means simultaneously, but a combination of lower attentional control (Basel et al., 2023) and the lack of explicit priming during the task hampered their ability to focus on global learning.

It is also possible that the OCD participants did not integrate the local information at all to estimate the global mean due to reduced confidence in their estimates. Ample previous research has found that OCD impairs confidence in one’s memory and learning (Vaghi et al., 2017; Wheaton et al., 2021; Morriss et al., 2018; Gagné & Radomsky 2020); compellingly, this reduced confidence in what one has learned has been found to link to compulsiveness scores, but not general psychiatric status or anxiety scores (Hermans et al., 2008), suggesting that it is indeed linked to the mechanism that drives formation of compulsions. Our current design did not allow participants to observe unlimited minions in any patch and possibly forced them to move on before they were confident that they’d learned an accurate canon position; this may have led them to not fully rely on their observations and inferences to estimate the global structure. One way to test this would be to remove the limitations on the number of arrow and the maximum number of minions from each canon; such alternative design might compensate for the low confidence in one’s local estimates enough to improve the integration into global estimates.

In relation to clinical symptoms, while OCD has been associated with higher doubt and reduced confidence in decision-making outside specific obsession-related checking behaviors (Nestadt et al., 2016), within their compulsive domains, it is not fully clear to what extent the increased uncertainty and doubt relate to current observations and outcomes, as opposed to the ability to generalize and accurately predict later outcomes. Previous models of OCD uncertainty have suggested that both excessive checking behaviors and the increased intolerance of uncertainty observed in OCD patients may result from higher uncertainty regarding both the current state and state transitions to future states (Fradkin et al., 2020). Our current evidence suggests it is rather the ability to predict the mechanism of state transitions, rather than the uncertainty about current state, that may underpin both phenomena. Thus, our data predict that patients are more likely to report uncertainty about the relationship between subgoals and global goals, rather than about the subgoals themselves (e.g. reporting doubt about whether they have cleaned their hands enough to avoid infection, rather than about whether their hands are clean; Gallagher, 2026). This may also be related to the threat overestimation observed both in physical and mental compulsions in OCD patients (Jacoby et al., 2018): while in threat overestimation, uncertainty about the local observations is relatively low (e.g. patients are aware of how long they have touched a contaminated object or which part of their clothes came into contact with it), the computation of the likelihood of later contamination is inaccurate. This suggests a need for deeper investigation into the extent to which current uncertainty reduction versus computations about future expected outcomes may be impaired in OCD.

## Conclusion

Using a hierarchical, predictive inference learning task that involves integrating global goals and local subgoals, we propose that compulsive behaviors associated with OCD are linked to deficits in uncertainty-processing; but, rather than a general failure in uncertainty reduction, OCD impacts the ability to recruit uncertainty pertaining to local subgoals in the service of reducing uncertainty about larger, more general goals. Such a deficit would reconcile existing accounts, by sparing local-level uncertainty reduction (which would show up as intact learning in a variety of tasks) but hampering goal-directed behavior in environments in which subgoals and goals coexist.
