# Supplementary material for: OCD Compulsions as Aberrant Integration of Uncertainty from Local Goals to Global Goals
Source: Comput Psychiatr. 2026 Aug 10;10(1):142–58. doi: 10.5334/cpsy.145 (PMC13475530; doi:10.5334/cpsy.145)
Supplement: Peer review history

# Reviewer report: Round 1, Reviewer A

**DOI:** 10.5334/cpsy.145.r1

**Authors:** Reviewer: A

## Recommendation:

Accept with Major Revision

## Comments/Explanation (required). Please include references to any minor or major revisions required, as well as addressing any issues with language clarity. Authors will see these comments:

Thank you for asking me to review this study. The authors offer an original computational account of OCD, focusing on the hierarchical integration and processing of goals. For this purpose, they have developed a novel task, wherein participants are expected to learn a hierarchically structured environment, while balancing of local and global parameter learning. Using this paradigm, they demonstrate a specific impairment in using global information to inform local predictions, a finding which they link to a purported clinical difficulty in informing high-level goals (e.g., stay healthy) based on the achievement of simple behavioral goals (e.g., clean hands).

The proposed hypothesis is novel and interesting, and the behavioral results supporting it are quite convincing. I still have several major concerns regarding the explanation of the computational model and the interpretation of the results, which I outline below

First, I found if somewhat difficult to properly evaluate the computational parts of the manuscript since some basic details were missing or assumed self-evident. Here are a few examples:

-The model seems to estimate σ^2^ based on its sample value (i.e., using a ‘known variance’ conjugate prior estimation), with no uncertainty. This restriction should be explicitly noted (along with the consequential assumption that uncertainty always decreases, which is not always the case when the variance is unknown)

-An explicit explanation for multiplying the mean of Equation 1 by σ_L0_^2^ (equations are numbered here by order, despite not being numbered in the original manuscript).

-Equation 2 is insufficiently explained. Please explain the derivation of σ^2^ (is it assumed equal across cannons and thereby aggregated?). Does σ_L0_^2^ affect Equation 2? how is \bar{\mu} calculated (is it averaged across cannons?)

-Equation 3 is also not properly explained, IMO. Please define T, and R, and explain the meaning of the normal distributions (does this pertain a random draw from a normal distribution? It’s CDF?)

-Did the authors use the Bayesian model for simulations and the RL model for fitting? How the two models were actually used in the manuscript is not explicitly explained. I also couldn’t find any information on how the RL model was fitted to the data.

-It would also be helpful if the authors could more explicitly (via simulations) demonstrate the correspondence (and misfits) between the Bayesian (nearly optimal) and heuristic (RL) model?

-Please explain how the hierarchical structure is implemented in the RL model (i.e., how do global estimates affect local estimates?). Thus, the authors explain how stay/leave decisions are affected by local and global prediction errors, but do not explain whether estimates (\mu) inform one another. Also, how (and when) are the global PEs updated in the RL model?

-Why is P(stay) in the RL model assumed binary vs. probabilistic? It seems strange given that PEs are random and after settling to average around zero, binarizing P(stay) could further emphasize noise.

Some of the behavioural analyses/results are also insufficiently clear:

-Please explain how local learning was measured separately from the effects of global priors. Was only the first cannon used for this analysis?

-What do accuracy units measure? i.e., how are the 0-100 or 0-1 scales related to the original units, which I assume to be something like deviation in cm?

-Figure 5A – please add confidence intervals.

-OCD patients and a model with no integration still seem showed improvement on the first trial of the second cannon –can the authors explain this effect?

Second, I found the theoretical basis and interpretation of the study somewhat insufficient.

-From a clinical perspective, patients often present uncertainty about subgoals (i.e., hands being clean), rather than only global goals. The theoretical connection to integration across time is interesting but requires further development and more explicit explanation.

-Some details emphasized in the introduction seem only remotely connected to the specific hypothesis in question. Indeed, the authors discuss uncertainty aversion, habit formation, and increases in OCD prevalence in recent years in the introduction, yet I do not see how the results do our could have contributed to these ideas. Similarly, in the discussion, the relevance to “oversampling/overfocusing” is unclear.

-In terms of interpretation, it seems that patients’ relative success in hitting the global goal despite not appearing to learn hierarchically requires further exploration. Indeed, the authors address this briefly in their discussion, where they say: “due to relatively small variances in the generative distributions, it is possible that they could still achieve accuracy on the spaceship trial by treating it as another ‘local’ game”. What I was missing is a computational treatment of this question – i.e., could a non-hierarchical model succeed in hitting the spaceship? Similarly, can the fitted model of patients explain their performance in finding the spaceship?

-The authors note that the Bayesian model “is expensive in computational and memory resources and requires perhaps more resources than participants are able or willing to expend on the task”. However, since it appears that the authors are using a conjugate prior type model, which only requires storing parameters (rather than distributions) I do not see why it is more expensive than an RL model.

Minor

-“The players earned a small reward (1 point) for accurately aiming arrows to hit each minion’s position” – I assume the authors meant to say “the cannon” position here?

# Reviewer report: Round 1, Reviewer B

**DOI:** 10.5334/cpsy.145.r2

**Authors:** Reviewer: B

## Recommendation:

Accept with Major Revision

## Comments/Explanation (required). Please include references to any minor or major revisions required, as well as addressing any issues with language clarity. Authors will see these comments:

In this manuscript, Geana et al. push the hypothesis that obsessive compulsive disorders (OCD) are associated with a deficit in the precision-dependent integration of local and global levels during hierarchical learning. To this end, they enrolled 20 patients with OCD and 20 controls to perform a gamified hierarchical learning task, and utilise computational models to formalise their theory and associated predictions.

While I found the general approach extremely interesting, exploring timely and exciting theories of OCD, and the presented results encouraging, the manuscript, as it stands, is not mature enough for publication. The computational approach needs to be more clearly articulated and leveraged, and the statistical analyses finessed, to convincingly substantiate the main claims of the manuscript. I have included some comments and suggestions below, which I hope will help the authors fully accomplish the great potential of this study.

## Sample

There are some inconsistencies in the description of the sample. For example, 55 out of 463 is 11.9%, not 8.4%. Additionally, a total of 12 participants have been excluded, which should leave 43 participants, not 40. Also, were the questionnaires administered to controls? It would be interesting to also report the levels of depression, anxiety, and intolerance to uncertainty in all participants, as they could be relatively high even in the general population.

## Task

The task description raised a few questions I would like the authors to clarify.

First, the task described in this manuscript is presented as an adaptation of a previous work (Genea et al, 2016) modified to now include a hierarchical structure. To my understanding, however, the task in the 2016 paper is almost identical, including the hierarchical structure and general gamification, and only differs on minor details such as the number of trials and exact payoffs. If critical differences have been introduced, they should be motivated and detailed in the manuscript, otherwise, the task should not be introduced as ‘novel’.

Second, and more importantly, I found it difficult to understand how exactly a trial played out and thus what the optimal strategy should be, and what behaviour was measured. On the one hand, the task description states that participants must aim at minions, whose location is drawn at each trial from a normal distribution that can encompass a large part of the screen, given the illustration 1.b. Accordingly, knowing the local mean, ie. the canon/commandant position, is only weakly predictive of the upcoming minion position and hitting one would require to shoot randomly in a general direction (unless participants could adjust their aim during the trial, which does not seem to be the case given the very blurry figure 1.a). In that case, I am not sure how to interpret the participant’s prediction as it will be largely impacted by some form of exploratory behaviour. On the other hand, a bit further down the manuscript, the authors state that an easy strategy is to aim at the mean of the previous observed minions, suggesting that hitting the canon/commandant - not the individual minions - is in fact the goal. While this would make more sense to interpret the participant’s anticipatory behaviour as a direct expression of their learning, it does not match the statements throughout the manuscript that the participant must aim at the minions. Could the authors clarify this point and explicitly state what their behavioural measure reflects?

In the same line, I am not sure how aiming at the global mean at the beginning of a new game is an optimal strategy. I do understand that this will minimize the expected prediction error, but if it does not increase the chances of hitting the next minion and therefore increase the chance of earning points, the participants have no incentive for doing so (more on that later).

Finally, as minor questions: Was the position of the archer or the angle of the arrow controlled by the participant? Was the prediction error calculated in pixels or in angular degrees? Did the participant get feedback (hit or miss, reward) on their performance? Could the screen asking for “stay” vs. “run” be included in figure 1 depicting the experiment?

## Modelling

The model presented here is quite interesting and seems adapted to the task. However, the description could be more didactic by progressively introducing, with a clear rationale, the different models and their constituting elements. As it is, the computational part is hard to follow and to relate to the (model-free) results. More precisely, my main concerns are that some core predictions of the models are not clearly motivated and explained (eg. the use of the general mean as prior for nested games) and that the competing model (reinforcement learning) is differing on too many points to allow a specific conclusion about the computational mechanism affected in OCD. I detail below some questions and suggestions that could help clarify the manuscript.

For the uncertainty-updating model, it would be useful to describe the basic elements of the model before dropping the update rules. For example, explicitize the assumption about the distributions (Gaussian seems the obvious guess, but still a guess). Also, if \bar{x} and sigma are time-dependent, why aren’t they indexed by t? How is \sigma computed exactly, in particular after the very first observation (one point is not enough), in order to compute the update? Why not use a Bayesian update to capture the evolution of the variance? More importantly, how did the authors come up with this update rule (provide mathematical rules, or reference if it was derived in a previous work)? For the global mean update, most of the notations are not even introduced (what is M_g, \bar{\mu}, etc.). How is \epsilon distributed? Where I got completely lost is when reaching the value function: what are T, R, \sigma_{G-1}? Were these values derived from first principles or did the authors come up with them to fit some intuition, as the text suggests? What aren’t they recursive, like the usual Bellman equations?

It would be helpful to clearly separate the model description per se (state evolution and observation equations, objective function) from how it is leveraged in the analysis. In particular, when the authors state that for the first game, the local mean is initialised randomly between 5 and 10, but then set to the global mean for the remaining game, it sounds like arbitrary choices made for simulations (in the end, nothing prevents from using the same simulation trick with the RL model...). However, the latter point is later used as a critical prediction of the model. The authors should carefully separate which prections are intrinsic to the model, and give the rationale in the model description (eg. a bayesian update is adaptive as it depends on variance) and which one pertains to simulation choices (eg. figure 3.A where the difference in the accuracy prediction could be due to ad hoc parametrisation).

More generally, it would be easier for the reader to know more in advance how the models will be used. I was initially expecting the models to be fitted to the data and formally compared, but it seems they are only used in simulations (at least for the Bayesian model) to derive qualitative predictions, which are in turn tested using classical (“model-free”) statistics. This is especially problematic as no details are given about the simulations (which parameter values for epsilon, gamma thresholds, learning rates, etc.; how many runs, and so on). Further, the models differ in many aspects (adaptive PE integration, precision weighted averaging of local means, global to local influence, precision dependent decision rule) and simply comparing the uncertainty updating model to the simple RL (which I assume is the one later refered to as the “no integration” model) is not a bit unfair. More importantly, the authors miss a wonderful opportunity to pinpoint what could be a fundamental issue of OCD in this task. For example, if one (wrongly) assumes that the variance of the observations is large, the Bayesian model should predict slower learning, less global updating, and longer stay times, in line with the empirical results. Failing to properly weight local estimates by their variance when computing the global mean would yield similar results (minus the slowed local learning). Introducing extra parameters in the Bayesian model to selectively impair sub-mechanisms, coupled to proper simulations, is required to properly dissociate those hypotheses. My point is that in order to claim, as done in the title, that OCD is selectively related to local to global integration deficit, comparing a full hierarchical Bayesian model to a flat RL model is not specific enough.

## Results

I am a bit puzzled by the argument that the influence of the global mean on each game’s initial trial is demonstrating an integration of local into global (beyond my criticism that the former is not well motivated in the model description). If anything, it shows an influence the other way around, that is, of the global onto the local. Testing the difference in accuracy on the final trial is however meaningful here. It would be interesting to push things further, though: does the accuracy depend on the number of canons seen, as expected? Are canons that had a larger end variance less influential than the ones precisely estimated, as predicted by the Bayesian model? To test this, the authors could eg. compare a linear model where the final aim is predicted by individual canon estimates weighted (vs not) by their estimated precision. This is critical as while OCD participants are indeed less accurate when aiming at the spaceship (a plot would be helpful here), they also observe fewer cannons as they stay longer in each game: the OCD group could have an intact (and bayesian) integration from local to global and their lower accuracy might be explained instead by some deficit in the decision rule which lead them to sample less (in other words, they are less accurate but not surprisingly so given they sampled fewer games).

Similarly, I do not entirely follow the results shown in Figure 3.C. Having shown figure 3.B that OCD participant have a lower global to local integration, I would have expected the learning deficit to mediate the lower accuracy (ie. all participants fall on a single regression line, but OCD have worse learning therefore bad terminal accuracy), not that the relationship between learning and accuracy to be broken. Could the author clarify how to interpret this correlation (or lack thereof)? Moreover, the authors should explicitly test for a difference between the groups (earning slope x group interaction): a difference in significance is not a significant difference.

Figure 3.B: showing standard error is necessary here, especially as I suspect that only a few OCD participants realised so many games. Model predictions should be shown only for the first trial of each game to match the x-axis and empirical data scale. How was the slope calculated? Is a linear regression really appropriate here? Also, shouldn’t the start of the plot (accuracy for the first minion) in figure 3.A be more or less aligned with the mean of the accuracy shown in 3.B?

Concerning the game duration analysis, how do the authors explain the lack of modulation in the OCD group? As both models predict an increase in duration with game number, the computational hypothesis of a deficit in local-global integration falls short of explaining the OCD behaviour. If anything, it shows that RL seems a better fit for the controls (the light grey line is very hard to see in figure 4.A). As mentioned above, an alternative hypothesis would be a problem in the decision rule (which itself could originate from a failure to learn the estimate precision). Again, the group x game interaction must be reported to conclude that OCD and controls differ; reporting that one group has a significant effect but not the other is insufficient.

The global-specific difference in learning rate is an interesting result, although it should be more explicitly tested (interaction group x condition) to be conclusive. I would however add this plot to the previous section as it relates to the local-global imbalance rather than stay/leave behaviour.

Concerning the attempt to deconstruct the strategy used to aim at the first minion of each game, I think that the glm approach is a good idea. However, lots of the regressors used here seem ad hoc (why 3 and 5 last, how was the weighting for AM?) and likely colinear and therefore unlikely to be significant. As suggested above, I would keep things simple (and orthogonal) and hypothesis oriented, eg.: last minion, mean of the last game except last minion, global mean excluding last game; then fit per subject to get some measure of variance about the estimates (or reconstruct it from a linear mixed effect model), then compare the two groups hoping to find that OCD participants rely only on local info while controls also use the global mean.

Finally, the dynamics of the aim variance are also interesting results. As it is unclear to me if this variance is strategic or just noise (see beginning of my review), I’m not sure how to interpret it. If strategic, I would try to relate this variance to individual trajectories reconstructed by the model: does it covary with local or global uncertainty? Are participants with a steeper decline in variance also the ones with better global learning? Larger modulation of stay time? Such analyses would be extremely helpful to untangle the relationship between the different layers of the model and how they translate into behavioural measures.

y

In the “local learning” section, was time entered as a continuous factor?

What is a two-way independent t-test? Do you mean two-sided unpaired?

Please mention which software / toolboxes have been used to simulate, fit, and conduct statistical analyses.

# Reviewer report: Round 2, Reviewer A

**DOI:** 10.5334/cpsy.145.r3

**Authors:** Reviewer: A

## Recommendation:

Accept with Minor Revision

## Comments/Explanation (required). Please include references to any minor or major revisions required, as well as addressing any issues with language clarity. Authors will see these comments:

The authors have done a commendable job addressing the previous rounds of feedback, particularly in clarifying the task mechanics and expanding on the clinical implications of the findings. The manuscript is stronger. However, there are a few remaining methodological and notational ambiguities, specifically within the computational modeling sections, that require clarification before publication.

1. Model Notation and Equations

- Equation 1 Inconsistencies: The explanation of Equation 1 contains several contradictory or undefined terms. The text states that the prior mean Mg is weighted by the prior local variance sigma2_L0, but the equation itself uses the initial global variance sigma2_G0.
- Undefined Variables: In Equation 1, sigma2_g,t is introduced but not explicitly defined in the text (presumably this is the posterior variance?). Similarly, please explicitly define sigma2_G,g–1 in Equation 2 and clarify the rationale for the g–1 index.
- Inconsistent Indexing: The text explaining Equation 1 suddenly introduces variables i and j (e.g., xi,j). Please define what these indices represent (e.g., trials within a game vs. game number) and ensure they match the g and t indices used in the other formulas. Furthermore, the posterior variance is denoted as sigma2_g,t in Equation 1, but appears to be referred to as sigma2_t in Equations 3 and 4. I would suggest to standardize the notation.
- Equations 3 and 4: The mathematical meaning of the term 1–N(0,σ2) remains unexplained. Please update this with the formal notation or explicitly define what this shorthand represents in the text.

2. Model Fitting and Mechanics

- RL Model Prediction Errors: It remains unclear exactly how and when ΔPE_G is calculated during the task. Given that global prediction errors update based on the discovery of new cannons, is ΔPE_G static during the course of a local game?
- Fitting Procedure: Please briefly clarify the objective function used for model fitting. Specifically, did the fitting procedure utilize a joint likelihood encompassing both the continuous aiming data and the discrete “stay/leave” decisions?

3. Terminology and Minor Corrections

- Leave vs. Run: The manuscript uses “Leave” and “Run” interchangeably.
- Model Justification: The text asserts that the Bayesian model is “computationally expensive” compared to the RL model. Given the use of simplified empirical priors, this (weakened) claim may still overstate the computational burden. I would suggest providing evidence for this claim or removing it.
- Typo: Page 21, Line 464: “bot excessive checking behaviors” should be “both”.

4. Supplemental Material

- The authors mention supplemental material, but I couldn’t find any in the materials I received.

# Reviewer report: Round 2, Reviewer B

**DOI:** 10.5334/cpsy.145.r4

**Authors:** Reviewer: B

## Recommendation:

Accept with Minor Revision

## Comments/Explanation (required). Please include references to any minor or major revisions required, as well as addressing any issues with language clarity. Authors will see these comments:

I would like to commend the authors on the work done for revising their manuscript. In particular, the description of the task now includes the pieces I thought were missing for proper understanding. While the model definition has also improved, there are, still, some (very) ambiguous notations that need to be clarifed, as I detail below. The provided text also suffers from several typos and mistakes that must be corrected before publication, which a careful proofreading would easily solve. Having said this, the rationale, experimental approach, and results are well constructed and highly interesting, and will make a great addition to the litterature once a few remaining issues, described in detail below, are adressed.

a) It seems that a couple of elements were involuntary cuts from the manuscript. Notably, Figure 2 is missing the legend for the panel D and E. More worrying, the discussion ends with an incomplete sentence (“In part this was constrained by practical considerations: while the computer screen for the task was relatively large, i”), suggesting a part of the text is simply missing. This looks like unfortunate mishap, which however prevents a complete evaluation of the work to be published.

b) My main issue with this otherwise compelling submission is the rather messy formalisation of the model. Numerous notations are inconsistent, unconventional, or simply not explained, which makes the mathematical derivation impossible to reproduce or implement (especially as the code doesn’t seem to be made publicly available). Given the importance of the computational approach both in the manuscript and the target journal, it is in my opinion necessary to tighten the equations before publication. In an attempt to stay constructive and expedite the review process, I will try to reformulate here my understanding of the model.

Using equation 1 to explain my point: the index i,j are not defined, but I understand them as g,t-1. \sigma_{L0} is nowhere to be found in the equation, and is defined as being both a prior (l.192) and inferred from previous process (l.218) suggesting some missing index (\sigma_{L,0} would make more sense). \sigma_{Local} looks like a variance but is in fact a sum of squares (SS) which needs to be normalised to make a variance estimate, which likely explain the $/t$ term (and I guess the j also); however, if this is the SS for all previous games, the normalisation should be g*T, not t. Using the notation \sigma_{Local} and \sigma_{L} for the observed and inferred variance is bit confusing.

I would expect a definition by recursion, something like this (where I dropped the explicit square so \sigma denotes the variance and not the standard deviation, using the exponent for indicating local/global):

- assuming the belief about the mothership position after the game g is $p(s_{g}) = N (\mu^G_{g}, \sigma^G_{g})$

- the (local) belief about the canon position $p(c_{g,t}|x_{1:t}) = N (\mu^L_{g,t}, \sigma^L_{g,t})$ is given for all trial t>0 by

$$\sigma^L_{g,t} = [1/\sigma^L_{g,0} + 1/\hat{\sigma^L_{g,t}}]^{-1}$$

$$\mu_{g,t} = \sigma^L_{g,t} [ \mu^G_{g-1} / \sigma^G_{g-1} + \hat{\mu_{g,t}}]$$

where $\hat{\mu_{g,t}}$ and $\hat{\sigma^L_{g,t}}$ are the mean and variance of the minion position up to t in the current game g (both set to 0 for t=0).

- When the game start (t=0) the prior local variance is defined by the free parameter $\sigma^L_{g,0}$. Notably, the prior local mean is then given by $(\sigma^L_{g,t}/ \sigma^G_{g-1}) \mu^G_{g-1}$, and thus explicitely depends on the current global belief.

A similar notation can then (possibly before) be used to describe the global belief update. Importantly, this clearly identify the values estimated from sensory inputs ($\hat{\mu_{g,t}}$ and $\hat{\sigma^L_{g,t}}$), and the ones defined by recursion of the Bayesian update rule, and more importantly the the free parameters of the model ($\mu^G_{0}$, $\sigma^G_{0}$, and $\sigma^L_{0}$). For the latter, the author could then explain how they were fitted to individual data, or sampled for the simulations.

Also, Line 207, what is \sigma ?

c) I could not make sense of the equations 3 and 4, despite a very clear description of the intent behind the conception of the policy (l.235-248). The values V are likely meant to be a real numbers (to be fed to the softmax). However, I (and likely other readers) find difficult to understand the terms in the form (1-N(0,\sigma)), suggesting random variables were used. Whether or not this is meant as a shorthand for some summary statistics of those distribution (which should be clarified), the meaning behind it would greatly benefit from some didactic effort.

d) Concerning the RL model, what are the free parameters? I see the two learning rates, initial values for local and global beliefs as good candidates. In which case, I’m not sure to see how the RL model could lose to the UU which has fewer parameters and a higher likelyhood (Figure 2.E).

More importantly, how were the integration/no-integration variants implemented? Was is by setting \mu_{g,0} to respectively \mu_{g-1,T} (global influence), or \mu_{0} (fixed prior)?

e) Figure 2 B and C greatly show that aiming at the local/global mean is a good strategy to hit minions (the ship), despite their randomness. Unfortunately, the legend of panel D and E are missing, but from what I understand:

- D: the LL is higher for the UU model than for the RL, suggesting that if the RL model wins the model selection, its due to its lower complexity. This point is worth mentioning (and emphasize the need to explicity list free parameters of the models).

- E: it seems that the model space is much larger that what the methods suggests. As most of those models are not described and do not win anyway, I suggest to limit the model space to only include conceptual hypotheses (RL without integration, RL with, UU).

f) Figure 3, which model was used for generating the predictions? Was it the integration and no-integration RL (justifying even more to explicitly explain their difference)?

g) In line 351-357, while I agree with the authors, an alternative interpretation is that while the global belief could in theory affect the ship accuracy in OCD, they fail to update this global belief from the beginning. In other words, the lack of correlation is not due to a different strategy, but by the fact the slope is alway zero (as suggested by Fig 3.B) and therefore cannot correlate with anything. Although this tension is well adressed in the discussion, this section of the result is a bit partial.

h) Figure 4.

- Despite using the same color code as in Figure 3, are the models used the same? And which version of the RL was used, with or without integration?

- The light gray is way to light to be seen on both paper and my computer screen, consider a darker shade.

- in the legend, the word “guard” is used instead of “minion”.

- diplaying the between group stats on the panel B would be helpful

i) In the discussion, the authors mention the screen could feasibly be split into 120 aim intervals (l.442). The methods section however suggests they were 200 intervals (l.159). Although a detail, this inconsistency should be fixed.

j) I think the sentence in parentheses l.176-177 was meant to be deleted. If not, there is for sure a typo as it’s unlikely participants performed 190 complete games on average.

# Reviewer report: Round 1, Author Response

**DOI:** 10.5334/cpsy.145.r5

Dear Editors,

Thank you for the opportunity to revise this manuscript. We have carefully addressed each comment raised by both reviewers, and believe the paper is considerably stronger as a result. A detailed point-by-point response is included below. We are hopeful that the revisions will be sufficient for acceptance and, given the extent to which we have engaged with all reviewer concerns, we would be grateful if you were able to assess the manuscript directly — though we fully understand if you feel it warrants another round of review.

Best regards,

Andra Geana

Reviewer Response Letter

We thank the reviewers for their insights and feedback, which have helped improve our manuscript significantly. We have revised our submission to address each reviewer’s concerns. We detail in the letter below our point-by-point responses to each. We appreciate the reviewers’ time and help.

Reviewer 1:

## Reviewer A:

-The model seems to estimate σ^2^ based on its sample value (i.e., using a ‘known variance’ conjugate prior estimation), with no uncertainty. This restriction should be explicitly noted (along with the consequential assumption that uncertainty always decreases, which is not always the case when the variance is unknown)

## Author’s response

We thank the reviewer for this point. We have made explicit note in the Modelling section of this assumption, as well as detailed our rationale for assuming that, given the generative structure of task rewards, the uncertainty around local and global means decreases with increased number of observations.

p.5, l.184-195: “The model tracked its own uncertainty about the estimated local means as the variance of the estimated local mean distribution, with no added noise. This assumption was considered reasonable in the context of the task given the relatively low generative variances and simple visual task demands: while adding noise to the estimated mean accurately reflected task performance, additional uncertainty in the estimates of sample variance was not considered to bias the decision significantly; thus the noise generally associated with imperfect Bayesian perceptual inference in humans (e.g. [51]) was integrated into our present model at the aim decision step, as noise parameter ε (see below).

With more observed samples (larger t in a game), that estimated uncertainty decreased as 1/*t*σ^2^ (see Figure 2A), and the probability of accurate aim on each subsequent trial increased (see Figure 2B). (While this is not always necessarily reflective of uncertainty in estimating generative variance, it was consistent with the task structure here, given the generative parameter settings).”

## Reviewer A:

-An explicit explanation for multiplying the mean of Equation 1 by σL02 (equations are numbered here by order, despite not being numbered in the original manuscript). Equation 2 is insufficiently explained. Please explain the derivation of $\sigma^{2}$ (is it assumed equal across cannons and thereby aggregated?). Does σL02 affect Equation 2? how is $\bar{\mu }$ calculated (is it averaged across cannons?)

## Author’s response

We thank the reviewer for pointing out the lack of clarity in these two model equations. We have properly numbered the equations and added further explanation of the assumption behind the derivations to the Modelling subsection.

p. 6, l.199-209. “For the model shown in Equations 1 and 2, rather than fully integrate we used a empirical Bayes approach to simplify the estimation: specifically, we assumed that for each new local process, the prior mean $\mu_{0}$ was drawn from the global process and the prior σL02 was inferred from the observations of all previous processes. Similarly, this pooled variance was used as an estimate for the hyperparameter σg2 (rather than maintain a probability distribution over the variance and marginalizing it out or using an MCMC sampler). The rationale for this simplification rested partially on the constraints on plausible mental computation and partially on keeping the model more easily adaptable to changes in task design that would require significant shifts in a fully-Bayesian inference. (e.g. adding multiple blocks with a similar hierarchical structure). The derivation of the equations (including the multiplication by σL02 ) fell out of these assumptions; we apologize for naming the posterior variance in a potentially confusing way given our notations about priors on mean/variances.”

We have reworked the notations in the equations perhaps more intuitively to


Equation 1 $$P\left(\mu_{g,t}|x_{t},x_{1:t–1}\right)=N\left(\sigma_{g,t}^{2}\left(\frac{M_{g}}{\sigma_{G0}^{2}}+\frac{j\cdot \bar{x_{i,j}}}{\sigma_{\text{Local}}^{2}}\right),{\left(\frac{1}{\sigma_{G0}^{2}}+\frac{1}{\sigma_{\text{Local}}^{2}/t}\right)}^{–1}\right),$$

Where $\bar{x_{i,j}}$ is the mean of the observed samples *x*_*i*1_, *x*_*i*2_, … *x_i,j_*, $\sigma_{L0}^{2}$ is the prior on local variance and $\sigma_{\text{Local}}^{2}$ the pooled sum of squared differences around the inferred means $\mu_{g,T}$ of all previous g1 games of length T.

For the global inference,


Equation 2 $$P\left(M_{g}|\mu_{g},\mu_{1:g–1}\right)=N\left(\sigma_{G,g–1}^{2}\left(\frac{M_{0}}{\sigma_{G0}^{2}}+\frac{\bar{\mu }}{\sigma_{\text{Local}}^{2}/g}\right),{\left(\frac{1}{\sigma_{G0}^{2}}+\frac{1}{\sigma_{G,g–1}^{2}/g}\right)}^{–1}\right)$$

Where $\bar{\mu }$ is indeed the inferred local means averaged across cannons, $m_{0}$ and $\sigma_{G0}^{2}$ are the initial priors on M, and $\sigma_{G,g–1}^{2}$ is the prior variance.

We have added the full derivation of both local and global mean inferences in a supplementary information document.

## Reviewer A:

-Equation 3 is also not properly explained, IMO. Please define T, and R, and explain the meaning of the normal distributions (does this pertain a random draw from a normal distribution? It’s CDF?)

## Author’s response

We thank the reviewer for pointing out the lack of clarity. We have further edited the modelling section to explain that T refers to the maximum trial duration of a game (calibrated to T = 30 in the present design), R is the reward obtained at the very end of the task for accurately hitting the overlord’s spaceship (i.e. correctly inferring the global generative mean; R was calibrated with a set of pilot studies and set to 30 points in the current design).

p.8, l.180-184: “For the first game, *μ*_0_ is initialized randomly to a position close to the middle of the screen (the same as the initial prior for the spaceship’s position, or the global mean), and $\sigma_{L0}^{2}$ is initialized randomly between 5 and 10. For the other games, the model uses estimates from the global mean distribution. The maximum game length, T, is set to 30 for this experiment; the local reward r is set to 1 point, and the global reward R is set to 30 points.”

## Reviewer A:

-Did the authors use the Bayesian model for simulations and the RL model for fitting? How the two models were actually used in the manuscript is not explicitly explained. I also couldn’t find any information on how the RL model was fitted to the data.

## Author’s response

We thank the reviewer for pointing out the lack of clarity here. We have edited the methods section to reflect which models were fit to the data and how.

Both models were used for simulation and fit to all participants’ data. Figure 2A shows the Bayesian model update process; we have added simulated learning curves for both models to Figure 2B and 2C; the predictions for local and global accuracy improving with increased numbers of observations are similar for both models, but occur through different underlying architectures (Bayesian inference vs. tracking trial-to-trial reductions in Prediction Error). Both models were fit to participants’ data, with the RL model fitting better in the majority of cases for both groups. We have added information to reflect this in the Modeling section, and added figures 2D and 2E to show the model fits.

## Reviewer A:

-It would also be helpful if the authors could more explicitly (via simulations) demonstrate the correspondence (and misfits) between the Bayesian (nearly optimal) and heuristic (RL) model?

## Author’s response

We thank the reviewer for this helpful suggestion. We have added simulated local and global learning curves for both models in Figure 2B and 2C to show the similar predictions of both models.

While both models predict increased local and global accuracy with increasing number of observations, their differ in their estimation of the optimal time for leaving a local game. The Bayesian model computes and compares estimated rewards for staying and leaving based on current uncertainty about the inferred local and global means (Equations 3 and 4); the RL model uses a threshold on the trial-by-trial local and global reductions in PE to decide whether an additional observation in the local game is more valuable than a new sample of the global distribution (Equations 8 and 9).

## Reviewer A:

-Please explain how the hierarchical structure is implemented in the RL model (i.e., how do global estimates affect local estimates?). Thus, the authors explain how stay/leave decisions are affected by local and global prediction errors, but do not explain whether estimates (\mu) inform one another. Also, how (and when) are the global PEs updated in the RL model?

## Author’s response

We thank the reviewer for pointing out this oversight. We have updated the methods section to reflect additional information on how the RL model integrates the local and global estimates.

p.12, l.270-274 “For this model, the global means were updated at the end of each game g. Multiple potential ways to include the learned global structure into local predictions were tested; based on model fits and in order to match the assumptions of the Bayes Uncertainty-Updating model, the current RL model similarly integrates the learned global structure into the aim decision of the first trial of each new game.”

## Reviewer A:

-Why is P(stay) in the RL model assumed binary vs. probabilistic? It seems strange given that PEs are random and after settling to average around zero, binarizing P(stay) could further emphasize noise.

## Author’s response

We fully agree that including an extra parameter in the RL model to account for a non-binary probability of leaving given the computed *ΔPEs* would yield a more flexible model. We have included a model comparison summary in Figure 2E that shows that the binary-decision model (with one fewer parameter) fit our sample better; that model was therefore chosen for inclusion in the methods section. While the probabilistic version with the extra parameter is more flexible and perhaps more intuitive, we suspect that the current pattern in model fits may have been due to the majority of participants (almost all in the OCD group, and several in the Healthy Controls group) did not choose to leave games; given that specific behavior in the current sample, it is likely that the binary stay/leave model performed better in model comparison due to having one less parameter.

## Reviewer A:

-Please explain how local learning was measured separately from the effects of global priors. Was only the first cannon used for this analysis?

## Author’s response

For the analysis shows in Figure 3, we did not separate the first cannon from the rest; all games were averaged out to show the local learning curves. We do agree with the reviewer’s observation that the first cannon would separate local learning mechanisms from the influence of global learning entirely; we now include that analysis in the Local Learning section of Results and have added an inline figure to Figure 3A to showcase the learning curves for only first game.

We did not separate the local games for this analysis as we did not seek to fully understand the influence of global priors on improving local estimates beyond the first observation of each game (that analysis targeting game-to-game changes in accuracy for the first observation was used as a measure of global learning, under the assumption that improvements in accuracy on the first trial were most heavily driven by global priors). However, the comparison of local learning performance did yield a mild difference between groups: we suspect that the mechanism for that mild difference lies with global-learning-induced improvements in accuracy in the Healthy Controls group. We now mention this assumption explicitly in the Discussion section.

p.12, l. 400-419 “It should be noted that while both groups learned the task at the local level, the OCD group may have shown some costs in the local learning performance of the lack of integration of global priors (Figure 3A). A potential mechanism for that small difference may be a benefit in the starting point of each new game associated with integrating new information about the global structure into the estimate of new local targets. However, the present task did not have sufficient data points to fully differentiate between that potential benefit of global learning on local trials and other mechanisms, such as increased noise, smaller/more precise trial-to-trial aim adjustments, memory decay or lower confidence in memory. While the potential differences in learning between the groups at the local level remain of interest, for the scope of this paper, we focused primarily on the binary distinction between improved learning on the local level and lack of evidence for global learning in the OCD group.”

## Reviewer A:

-What do accuracy units measure? i.e., how are the 0-100 or 0-1 scales related to the original units, which I assume to be something like deviation in cm?

## Author’s response

We apologize for the lack of clarity; we have added detail to the Methods section explaining how accuracy is measured.

p.7, l. 158-167. “Players obtained a reward based on the accuracy of each trial’s aim. Accuracy was measured by the difference in y-axis position between the player’s aim and the minion’s position; the y-axis was split into 200 intervals measured relative to the size of the experimental screen (so in relative screen size units rather than absolute centimeters or pixels; all participants completed the task on the same screen, so they all experienced the same accuracy calculations. The task was designed so a ‘hit’ on screen occurred if the aim deviation from the minion position fell below the standard deviation of the local generative distribution *σ_Local_*. This coincided with a small window of about 25px around each minion’s y-axis position. Given the task structure, the chance for earning rewards in each game was indeed maximized by aiming at the position of the local cannon – but participants only earned points if they actually “hit” the minion.”

## Reviewer A:

-Figure 5A – please add confidence intervals.

## Author’s response

We thank the reviewer for this suggestion; confidence intervals have been added to the figure.

## Reviewer A:

-OCD patients and a model with no integration still seem showed improvement on the first trial of the second cannon –can the authors explain this effect?

## Author’s response

We thank the reviewer for raising this point. We did indeed notice this effect as well and we have now added a brief section addressing it under Discussion. We assume that the improvement on the second game (first trial of the second canon) in the OCD group is likely due to an anchoring-like effect from the last trial (or the last few trials) of the previous game; as there is no knowledge on the first trial of the first game, participants aim randomly and are often quite far from the true mean; on the second game, they are more likely to aim close to the previous trials. We have previously run analyses to predict whether the aim on the first trial in the new game for the OCD group was best predicted by the learned game mean (“local” mean in the model), the last trial of a game, a moving window of the last few trials, a (correct or incorrect) trial at random from the previous game, or similar factors. Those analyses were inconclusive, with perhaps the strongest evidence in the direction of a general sampling of a random number in the range of the previous observations. (By contrast, the healthy volunteers group aim was more strongly predicted by the local mean and the last few trials –which, due to the structure of the task, were often highly correlated with the mean).

## Reviewer A:

-From a clinical perspective, patients often present uncertainty about subgoals (i.e., hands being clean), rather than only global goals. The theoretical connection to integration across time is interesting but requires further development and more explicit explanation.

## Author’s response

We thank the reviewer for this point. We have added a paragraph in the Discussion addressing in more detail the extent to which clinical presentation is related to uncertainty about subgoals vs. broader uncertainty about the extent to which observed subgoals are incorporated into bigger/more relevant goals that produce more uncertainty.

p. 21, l.461-475 “In relation to clinical symptoms, while OCD has been associated with higher doubt and reduced confidence in decision-making outside specific obsession-related checking behaviors [51], within their compulsive domains, it is not fully clear to what extent the increased uncertainty and doubt relate to current observations and outcomes, as opposed to the ability to generalize and accurately predict later outcomes. Previous models of OCD uncertainty have suggested that bot excessive checking behaviors and the increased intolerance of uncertainty observed in OCD patients may result from higher uncertainty regarding both the current state and state transitions to future states [25]. Our current evidence suggests it is rather the ability to predict the mechanism of state transitions, rather than the uncertainty about current state, that may underpin both phenomena. Thus, our data predict that patients are more likely to report uncertainty about the relationship between subgoals and global goals, rather than about the subgoals themselves (e.g. reporting doubt about whether they have cleaned their hands enough to avoid infection, rather than about whether their hands are clean; [52]). This may also be related to the threat overestimation observed both in physical and mental compulsions in OCD patients [53]: while in threat overestimation, uncertainty about the local observations is relatively low (e.g. patients are aware of how long they have touched a contaminated object or which part of their clothes came into contact with it), the computation of the likelihood of later contamination is inaccurate. This suggests a need for deeper investigation into the extent to which current uncertainty reduction versus computations about future expected outcomes may be impaired in OCD.”

## Reviewer A:

-Some details emphasized in the introduction seem only remotely connected to the specific hypothesis in question. Indeed, the authors discuss uncertainty aversion, habit formation, and increases in OCD prevalence in recent years in the introduction, yet I do not see how the results do our could have contributed to these ideas. Similarly, in the discussion, the relevance to “oversampling/overfocusing” is unclear.

## Author’s response

We thank the reviewer for pointing out the lack of clarity and connection. We hope that edits to the Introduction and Discussion have removed irrelevant references and clarified the connection to oversampling.

## Reviewer A:

-In terms of interpretation, it seems that patients’ relative success in hitting the global goal despite not appearing to learn hierarchically requires further exploration. Indeed, the authors address this briefly in their discussion, where they say: “due to relatively small variances in the generative distributions, it is possible that they could still achieve accuracy on the spaceship trial by treating it as another ‘local’ game”. What I was missing is a computational treatment of this question – i.e., could a non-hierarchical model succeed in hitting the spaceship? Similarly, can the fitted model of patients explain their performance in finding the spaceship?

## Author’s response

We thank the reviewer for this excellent point. A non-hierarchical model can still hit the spaceship at the end of the game, though with a lower probability, as the spaceship position is still linked to the observed positions of all minions in the game. Given that minions were randomly sampled for all participants, and the relatively low number of games, it is possible that some participants had an easier time hitting the spaceship through non-hierarchical strategies (e.g. if the final canon, or the final minion happened to be close to the spaceship position). Overall, however, the probability of hitting the global mean is maximized by using information about the hierarchical structure to integrate local means into a global mean estimate.

We have added more information in the Discussion to address this point. “The practicality of maintaining a meaningful dependency between minion waves while sufficiently separating the waves visually on the computer screen, while at the same time keeping all stimuli sufficiently large as to remain accessible and engaging for the players, poses a potential limitation to the study design. As the screen could feasibly be split into about 120 aim intervals while preserving reasonable visibility in the game, the design question of calibrating variances, minion location constraints, minion sizes and other design parameters was carefully considered and tested over several small-scale pilot studies to calibrate these to the best possible extent to suit our goal of comparing two strategies: one that takes into account learned information about the global structure of rewards (and thus optimally aims at the current estimated global mean), and one that does not. For the latter, several aim strategies may be included, as discussed above: aiming at random, aiming at the last minion, and other options as shown in Figure 5A. While the relatively low number of games per participant and the design constraints made it more challenging to fully compute the cost of suboptimal strategies (partly due to the fact that it was still possible – though less likely – to hit the spaceship on the final trial without uncertainty integration), the strategy of aiming at the current inferred mean on the first trial of each new game remains mathematically optimal, and, we argue, intuitive in the context of the task’s narrative story. ”

## Reviewer A:

-The authors note that the Bayesian model “is expensive in computational and memory resources and requires perhaps more resources than participants are able or willing to expend on the task”. However, since it appears that the authors are using a conjugate prior type model, which only requires storing parameters (rather than distributions) I do not see why it is more expensive than an RL model.

## Author’s response

We thank the reviewer for pointing out this inconsistency. It is correct that we intentionally simplified the Bayesian model as much as possible while preserving the core inference processes, in order to address the plausibility of computation issue. We have modified the above phrase to more accurately reflect the increased computation needs of Bayesian inference compared to RL, while removing the less accurate claim about increased memory resources.

## Reviewer A:

Minor

-“The players earned a small reward (1 point) for accurately aiming arrows to hit each minion’s position” – I assume the authors meant to say “the cannon” position here?

## Author’s response

We apologize for the lack of clarity and have corrected the Methods text to more accurately reflect the gameplay rules. Players earned a small reward (1 point) for aiming arrows within a small window of about 25px around each minion’s y-axis position; given the task structure, the chance for earning rewards in each game was indeed maximized by aiming at the position of the local cannon – but participants only earned points if they actually “hit” the minion (with a “hit” happening if they aimed within that small window around the minion’s central position).

## Reviewer B:

## Sample

There are some inconsistencies in the description of the sample. For example, 55 out of 463 is 11.9%, not 8.4%. Additionally, a total of 12 participants have been excluded, which should leave 43 participants, not 40. Also, were the questionnaires administered to controls? It would be interesting to also report the levels of depression, anxiety, and intolerance to uncertainty in all participants, as they could be relatively high even in the general population.

## Author’s response

We thank the reviewer for pointing out the inconsistencies in the reported numbers. We have corrected the errors in the Participants subsection to accurately reflect the recruitment and participant inclusion process and final sample size. Clinical questionnaires were not administered to the control group; we agree with the reviewer that comparing levels of depression, anxiety, and especially intolerance of uncertainty hold potential relevance here. Unfortunately the data is not fully available for analysis in this sample.

## Reviewer B:

## Task

First, the task described in this manuscript is presented as an adaptation of a previous work modified to now include a hierarchical structure. To my understanding, however, the task in the 2016 paper is almost identical, including the hierarchical structure and general gamification, and only differs on minor details such as the number of trials and exact payoffs. If critical differences have been introduced, they should be motivated and detailed in the manuscript, otherwise, the task should not be introduced as ‘novel’.

## Author’s response

We thank the reviewer for raising this point. Indeed, the 2016 version of the task was conceptualized around a similar idea of trading off immediate small reward with information relevant to later large rewards, and gamified the problem in a similar way. The changes to the present task may have appeared critical to us from a conceptualization and modelling standpoint, but indeed may not warrant the use of the word “novel”. We have replaced that with the more accurate description of the task as an “updated design”. We have also detailed differences from the original task version: the changes include different underlying generative distributions (increasing the range of generative means and increasing the variances) and task parameters (total number of steps and the final reward were increased), as well as visual changes in the task that reduced reliance on memory and perception ( more visible hits, longer times to observe arrow aims, and an on-screen counter of hits/misses per game, as well as overall arrows.)

## Reviewer B:

Second, and more importantly, I found it difficult to understand how exactly a trial played out and thus what the optimal strategy should be, and what behaviour was measured. On the one hand, the task description states that participants must aim at minions, whose location is drawn at each trial from a normal distribution that can encompass a large part of the screen, given the illustration 1.b. Accordingly, knowing the local mean, ie. the canon/commandant position, is only weakly predictive of the upcoming minion position and hitting one would require to shoot randomly in a general direction (unless participants could adjust their aim during the trial, which does not seem to be the case given the very blurry figure 1.a). In that case, I am not sure how to interpret the participant’s prediction as it will be largely impacted by some form of exploratory behaviour. On the other hand, a bit further down the manuscript, the authors state that an easy strategy is to aim at the mean of the previous observed minions, suggesting that hitting the canon/commandant - not the individual minions - is in fact the goal. While this would make more sense to interpret the participant’s anticipatory behaviour as a direct expression of their learning, it does not match the statements throughout the manuscript that the participant must aim at the minions. Could the authors clarify this point and explicitly state what their behavioural measure reflects?.

## Author’s response

We apologize for the lack of clarity here. We have updated the Methods section to reflect a more detailed and hopefully clearer description of how aim and performance were correlated in the task.

p.7, 158-167 “Players obtained a reward based on the accuracy of each trial’s aim. Accuracy was measured by the difference in y-axis position between the player’s aim and the minion’s position; the y-axis was split into 200 intervals measured relative to the size of the experimental screen (so in relative screen size units rather than absolute centimeters or pixels; all participants completed the task on the same screen, so they all experienced the same accuracy calculations. The task was designed so a ‘hit’ on screen occurred if the aim deviation from the minion position fell below the standard deviation of the local generative distribution *σ_Local_*. This coincided with a small window of about 25px around each minion’s y-axis position. Given the task structure, the chance for earning rewards in each game was indeed maximized by aiming at the position of the local cannon – but participants only earned points if they actually “hit” the minion.”

## Reviewer B:

In the same line, I am not sure how aiming at the global mean at the beginning of a new game is an optimal strategy. I do understand that this will minimize the expected prediction error, but if it does not increase the chances of hitting the next minion and therefore increase the chance of earning points, the participants have no incentive for doing so (more on that later)

## Author’s response

We agree that the decision of where to optimally aim at the beginning of the new game is not straightforward. In our current paper, we compare primarily two strategies: one that takes into account learned information about the global structure of rewards, and one that does not. Under this framework, aiming at the global mean does increase the chance of hitting the next minion compared to the non-integrating strategies (which include aiming at random, aiming at the last observed minion, or – to a lesser extent – aiming at the mean of the last few minions). It is true that the variances in local and global generative processes here were relatively small and thus simplified the problem of aiming in practice for our participants. In part this was constrained by practical considerations: while the computer screen for the task was relatively large, it could still only feasibly be split into about 120 potential aim intervals while preserving reasonable visibility in the game. The design question of calibrating variances so as to preserve a clear connection between cannons while also ensuring that each local wave of minions was relatively differentiable from other local waves was carefully considered, and we ran several small-scale pilot studies to calibrate these design parameters to the best possible extent. Ultimately, we agree with the reviewer that the practical dependencies between the locations of minions in different waves make it difficult to fully separate all potential strategies for the first aim in each new game. Nonetheless, under the existing constraints of the design and collected data, aiming at the global mean is both the mathematically optimal decision on the first trial of each game, and the intuitive choice.

We now address this explicitly in the Discussion section. “The practicality of maintaining a meaningful dependency between minion waves while sufficiently separating the waves visually on the computer screen, while at the same time keeping all stimuli sufficiently large as to remain accessible and engaging for the players, poses a potential limitation to the study design. As the screen could feasibly be split into about 120 aim intervals while preserving reasonable visibility in the game, the design question of calibrating variances, minion location constraints, minion sizes and other design parameters was carefully considered and tested over several small-scale pilot studies to calibrate these to the best possible extent to suit our goal of comparing two strategies: one that takes into account learned information about the global structure of rewards (and thus optimally aims at the current estimated global mean), and one that does not. For the latter, several aim strategies may be included, as discussed above: aiming at random, aiming at the last minion, and other options as shown in Figure 5A. While the relatively low number of games per participant and the design constraints made it more challenging to fully compute the cost of suboptimal strategies (partly due to the fact that it was still possible – though less likely – to hit the spaceship on the final trial without uncertainty integration), the strategy of aiming at the current inferred mean on the first trial of each new game remains mathematically optimal, and, we argue, intuitive in the context of the task’s narrative story.”

## Reviewer B:

Finally, as minor questions: Was the position of the archer or the angle of the arrow controlled by the participant? Was the prediction error calculated in pixels or in angular degrees? Did the participant get feedback (hit or miss, reward) on their performance? Could the screen asking for “stay” vs. “run” be included in figure 1 depicting the experiment?

## Author’s response

We thank the reviewer for pointing out the lack of clarity here. We have included additional explanation in the Task section of Methods, and changed Figure 1 to include visual feedback on ‘hits’ as well as the on-screen hit counters.

In this version of the archer task, the position of the archer was controlled by the participant using the mouse. The prediction error was calculated in pixels, and the participant obtained visual feedback (the minion ‘exploded’ if hit by the arrow, and the arrow flew past the minion to the end of the screen if not) and observed increases in the ‘hit’ count after each hit and in the ‘miss’ count after each miss. There was no screen asking for “stay” vs. “run” – the “run” button was available on screen at all times, and participants could click it after each trial in order to end the current game and start a new one.

## Reviewer B:

## Modelling

The model presented here is quite interesting and seems adapted to the task. However, the description could be more didactic by progressively introducing, with a clear rationale, the different models and their constituting elements. As it is, the computational part is hard to follow and to relate to the (model-free) results. More precisely, my main concerns are that some core predictions of the models are not clearly motivated and explained (eg. the use of the general mean as prior for nested games) and that the competing model (reinforcement learning) is differing on too many points to allow a specific conclusion about the computational mechanism affected in OCD. I detail below some questions and suggestions that could help clarify the manuscript.

For the uncertainty-updating model, it would be useful to describe the basic elements of the model before dropping the update rules. For example, explicitize the assumption about the distributions (Gaussian seems the obvious guess, but still a guess). Also, if $\bar{x}$ and sigma are time-dependent, why aren’t they indexed by t? How is \sigma computed exactly, in particular after the very first observation (one point is not enough), in order to compute the update? Why not use a Bayesian update to capture the evolution of the variance? More importantly, how did the authors come up with this update rule (provide mathematical rules, or reference if it was derived in a previous work)? For the global mean update, most of the notations are not even introduced (what is $M_{g},\bar{\mu }$ , etc.). How is ϵ distributed? Where I got completely lost is when reaching the value function: what are T, R, $\sigma_{G–1}$ ? Were these values derived from first principles or did the authors come up with them to fit some intuition, as the text suggests? What aren’t they recursive, like the usual Bellman equations?

## Author’s response

We apologize for the lack of clarity and thank the reviewer for these excellent suggestions. We have edited the Uncertainty-Updating model section to explicitly state that the distributions are Gaussian, to define all terms in the equations including M, g, T, R, and to show the proper time indexing in the model equations.

Regarding variance estimation in the Bayesian model, we did simplify the assumptions there rather than use the full Bayesian inference of variance. We have clarified our assumptions and the rationale for these assumptions in the Modeling section.

p. 6, l.199-209. “For the model shown in Equations 1 and 2, rather than fully integrate we used a empirical Bayes approach to simplify the estimation: specifically, we assumed that for each new local process, the prior mean $\mu_{0}$ was drawn from the global process and the prior σL02 was inferred from the observations of all previous processes. Similarly, this pooled variance was used as an estimate for the hyperparameter σg2 (rather than maintain a probability distribution over the variance and marginalizing it out or using an MCMC sampler). The rationale for this simplification rested partially on the constraints on plausible mental computation and partially on keeping the model more easily adaptable to changes in task design that would require significant shifts in a fully-Bayesian inference. (e.g. adding multiple blocks with a similar hierarchical structure). The derivation of the equations (including the multiplication by σL02 ) fell out of these assumptions; we apologize for naming the posterior variance in a potentially confusing way given our notations about priors on mean/variances.”

## Reviewer B:

It would be helpful to clearly separate the model description per se (state evolution and observation equations, objective function) from how it is leveraged in the analysis. In particular, when the authors state that for the first game, the local mean is initialised randomly between 5 and 10, but then set to the global mean for the remaining game, it sounds like arbitrary choices made for simulations (in the end, nothing prevents from using the same simulation trick with the RL model...). However, the latter point is later used as a critical prediction of the model. The authors should carefully separate which prections are intrinsic to the model, and give the rationale in the model description (eg. a bayesian update is adaptive as it depends on variance) and which one pertains to simulation choices (eg. figure 3.A where the difference in the accuracy prediction could be due to ad hoc parametrisation). More generally, it would be easier for the reader to know more in advance how the models will be used. I was initially expecting the models to be fitted to the data and formally compared, but it seems they are only used in simulations (at least for the Bayesian model) to derive qualitative predictions, which are in turn tested using classical (“model-free”) statistics. This is especially problematic as no details are given about the simulations (which parameter values for epsilon, gamma thresholds, learning rates, etc.; how many runs, and so on). Further, the models differ in many aspects (adaptive PE integration, precision weighted averaging of local means, global to local influence, precision dependent decision rule) and simply comparing the uncertainty updating model to the simple RL (which I assume is the one later refered to as the “no integration” model) is not a bit unfair. More importantly, the authors miss a wonderful opportunity to pinpoint what could be a fundamental issue of OCD in this task. For example, if one (wrongly) assumes that the variance of the observations is large, the Bayesian model should predict slower learning, less global updating, and longer stay times, in line with the empirical results. Failing to properly weight local estimates by their variance when computing the global mean would yield similar results (minus the slowed local learning). Introducing extra parameters in the Bayesian model to selectively impair sub-mechanisms, coupled to proper simulations, is required to properly dissociate those hypotheses. My point is that in order to claim, as done in the title, that OCD is selectively related to local to global integration deficit, comparing a full hierarchical Bayesian model to a flat RL model is not specific enough.

## Author’s response

We thank the reviewer for this point and agree that there is significant value in delving more deeply into the implications of distinct model mechanisms and further changing the models to better understand potential predictions for optimal strategies and learning performance that stem from those distinct mechanisms. In the present paper, while we did fit models to our participants data, the reviewer is entirely correct that the main goal of our two presented models was to show that, under two different potential decision architectures (one involving Bayesian optimal uncertainty updating, albeit with a simplified variance assumption, and the other relying on trial-and-error and tracking reductions in PE), a model that integrates information across games outperforms a no-integration model, and that the model predictions in both cases resemble the Healthy Volunteers group behavior and not the Obsessive Compulsive group. While we tested several potential models for both RL and Bayesian architectures, we ultimately selected the two presented in the paper both due to slightly better fit (although not all participants, as expected, were best fit by the same model) but more so – as the reviewer correctly states – to the value of establishing an intuition about potential ways to integrate information from local subgoals to global goals.

We fully agree that there is much more to be learned by examining more deeply potential model differences and their predictions; this makes the object of future work, but remains outside the scope of the current paper, whose main goal is to showcase the behavioral evidence of impaired integration in OCD. To that goal, the models presented here – mainly via simulation – showcase the theory behind our proposed mechanism, without detailing specific assumptions about which model is better fit to either participant group…

## Reviewer B:

## Results

I am a bit puzzled by the argument that the influence of the global mean on each game’s initial trial is demonstrating an integration of local into global (beyond my criticism that the former is not well motivated in the model description). If anything, it shows an influence the other way around, that is, of the global onto the local. Testing the difference in accuracy on the final trial is however meaningful here. It would be interesting to push things further, though: does the accuracy depend on the number of canons seen, as expected? Are canons that had a larger end variance less influential than the ones precisely estimated, as predicted by the Bayesian model? To test this, the authors could eg. compare a linear model where the final aim is predicted by individual canon estimates weighted (vs not) by their estimated precision. This is critical as while OCD participants are indeed less accurate when aiming at the spaceship (a plot would be helpful here), they also observe fewer cannons as they stay longer in each game: the OCD group could have an intact (and bayesian) integration from local to global and their lower accuracy might be explained instead by some deficit in the decision rule which lead them to sample less (in other words, they are less accurate but not surprisingly so given they sampled fewer games).

We thank the reviewer for this interesting suggestion. The accuracy on the final trial does indeed depend on the number of cannons seen, at least in simulation. In the practice, the number of cannons seen for the OCD group is nearly identical (as the majority of the group did not leave games or, when they did, they left after 25+ trials, and thus only had one extra game/cannon), and, while the number of cannons seen for the Healthy Volunteers shows a bit more variability, there is still not enough to draw significant conclusions. Simulations however do support the reviewer’s intuition; we show that in Figure 2C.

## Author’s response

The differential impact of cannons on the final accuracy is an extremely interesting suggestion. Testing it in our current dataset is slightly complicated by the fact that inferred variance for each cannon depends on the number of minions observed in the cannon, and the OCD group consistently oversampled locally, thus observing on average about 9 more minions than the control group. However, the overall number of cannons sampled between the two groups is not different enough under our current design parameters to explain the lower global accuracy in the OCD group. (While the control group did sample more cannons, it was usually 2-3 more, which, given the relatively low variances and the relatively high # cannons already sampled, should not significantly improve accuracy.)

## Reviewer B:

Similarly, I do not entirely follow the results shown in Figure 3.C. Having shown figure 3.B that OCD participant have a lower global to local integration, I would have expected the learning deficit to mediate the lower accuracy (ie. all participants fall on a single regression line, but OCD have worse learning therefore bad terminal accuracy), not that the relationship between learning and accuracy to be broken. Could the author clarify how to interpret this correlation (or lack thereof)? Moreover, the authors should explicitly test for a difference between the groups (earning slope x group interaction): a difference in significance is not a significant difference.

## Author’s response

We thank the reviewer for pointing out the need for further interpretation here. We have clarified that section to include our interpretation that a correlation between global learning and spaceship accuracy makes sense under the assumption that estimated global means play a role in determining aim on the final (spaceship) trial. Absent that assumption, we would expect no correlation between the two, as in our current data: here, it appears that the majority of the OCD group likely used a different strategy for the final aim. As per our earlier discussion, given the design parameters and the sample size, it is difficult to precisely conclude what strategy the OCD participants may be using; however, several strategies, including random aim, aiming at the last observed minion, a noisy mean of all observed minions, and others, in addition to potential variability among participants in their chosen strategy, would decouple the global learning from the final trial accuracy, as we observe here.

“It is worth nothing that this correlation between global learning and spaceship accuracy makes sense under the assumption that estimated global means play a role in determining aim on the final (spaceship) trial. Absent that assumption, we would expect no correlation between the two. Strategies that do not integrate learned information about the global mean into the final aim – including random aim, aiming at the last observed minion, a noisy mean of all observed minions, and others – in addition to potential variability among participants in their chosen strategy, would decouple the global learning from the final trial accuracy.”

## Reviewer B:

Concerning the game duration analysis, how do the authors explain the lack of modulation in the OCD group? As both models predict an increase in duration with game number, the computational hypothesis of a deficit in local-global integration falls short of explaining the OCD behaviour. If anything, it shows that RL seems a better fit for the controls (the light grey line is very hard to see in figure 4.A). As mentioned above, an alternative hypothesis would be a problem in the decision rule (which itself could originate from a failure to learn the estimate precision). Again, the group x game interaction must be reported to conclude that OCD and controls differ; reporting that one group has a significant effect but not the other is insufficient.

## Author’s response

We have updated the Results section to report the interaction. It is accurate that the RL model is a better fit for the control group participants – both the RL and the Uncertainty-Updating Model assume integration of global information into decisions on local games, which is what we observe in the control group. While it is true that while the Bayesian model fit some control group participants better, the majority were fit better by the RL model with the integration assumption.

## Reviewer B:

The global-specific difference in learning rate is an interesting result, although it should be more explicitly tested (interaction group x condition) to be conclusive. I would however add this plot to the previous section as it relates to the local-global imbalance rather than stay/leave behaviour. Concerning the attempt to deconstruct the strategy used to aim at the first minion of each game, I think that the glm approach is a good idea. However, lots of the regressors used here seem ad hoc (why 3 and 5 last, how was the weighting for AM?) and likely colinear and therefore unlikely to be significant. As suggested above, I would keep things simple (and orthogonal) and hypothesis oriented, eg.: last minion, mean of the last game except last minion, global mean excluding last game; then fit per subject to get some measure of variance about the estimates (or reconstruct it from a linear mixed effect model), then compare the two groups hoping to find that OCD participants rely only on local info while controls also use the global mean.

## Author’s response

We are thankful for the reviewer’s suggestions here. We agree that the learning rate plot associates well with the local-global imbalance; our original intent for placing it in the figure next to the Stay/Leave plot was to emphasize our hypothesized connection between global learning and stay times. We are keeping the plot there for this reason, though we agree that it can also fit very well in the previous figure.

The GLM terms showing a moving window for the last few minions relates to a potential mechanism of reduced working memory capacity in OCD hampering local integration. We also fit a decay term into our models (though that term did not improve fit for either model) to test the same assumption. The weighted mean reported in the same GLM was also a reflection of this memory decay assumption, and was weighted more heavily toward recently observed minions (although several other weighing functions were also fit, with no observable benefit, and thus left out of the reported analysis.)

## Reviewer B:

Finally, the dynamics of the aim variance are also interesting results. As it is unclear to me if this variance is strategic or just noise (see beginning of my review), I’m not sure how to interpret it. If strategic, I would try to relate this variance to individual trajectories reconstructed by the model: does it covary with local or global uncertainty? Are participants with a steeper decline in variance also the ones with better global learning? Larger modulation of stay time? Such analyses would be extremely helpful to untangle the relationship between the different layers of the model and how they translate into behavioural measures.

## Author’s response

We thank the reviewer for the suggestion of additional analyses here. We did investigate the relationship between decline in aim variance and global learning; we have now included the results of this analysis showing that indeed participants with better global learning also adjust their aim less later in the game.

Unfortunately, due to the relatively low variability in stay times (with almost none of the OCD group leaving games early) we were not able to meaningfully compare the groups for potential relationships between stay time and global learning.

## Reviewer B:

In the “local learning” section, was time entered as a continuous factor?

## Author’s response

We have clarified in the Local Learning section that Time was entered as a discrete factor (trial number).

## Reviewer B:

What is a two-way independent t-test? Do you mean two-sided unpaired?

## Author’s response

We apologize for the lack of clarity in phrasing. We ran a two-tailed independent samples t-test here. We have changed the Results phrasing to reflect that.

## Reviewer B:

Please mention which software / toolboxes have been used to simulate, fit, and conduct statistical analyses.

## Author’s response

We thank the reviewer for pointing out this oversight. We fit all models and ran all statistical analyses in MATLAB, using the Statistics and Machine Learning toolbox and have clarified the Methods section to include this.
